# Rewriting the viral script: post-translational modifications orchestrating SARS-CoV-2 pathogenesis and immune evasion

**DOI:** 10.3389/fmicb.2026.1748470

**Published:** 2026-02-10

**Authors:** Jie Qu, Minglong Liu, Chen Zhou

**Affiliations:** 1 Department of Cadre Ward, The First Hospital of Jilin University, Changchun, China; 2 General Department, The First Hospital of Jilin University (The Eastern Division), Changchun, China

**Keywords:** host–virus interactions, post-translational modifications, SARS-CoV-2 pathogenesis, therapeutic targeting, viral immune evasion

## Abstract

SARS-CoV-2 reprograms host cell biology not solely through its genomic content but also through a sophisticated arsenal of post-translational modifications (PTMs) that modulate viral protein function, host signaling networks, and immune responses. Despite increasing recognition of PTMs as dynamic regulators of infection, their full functional breadth and therapeutic potential remain incompletely defined. Here, we provide a comprehensive, PTM-centric synthesis of SARS-CoV-2 pathogenesis, detailing how phosphorylation, ubiquitination, SUMOylation, glycosylation, acetylation, succinylation, ISGylation, and ADP-ribosylation cooperatively shape virus–host interplay. We dissect the mechanistic roles of individual modifications, such as phosphorylation-mediated transitions in nucleocapsid function, ubiquitin-driven degradation of immune factors, and SUMOylation-guided viral assembly, while revealing higher-order regulatory circuits and crosstalk among PTMs. Additionally, we highlight emerging computational tools for PTM site prediction and identify shared enzymatic nodes exploitable for host-directed antiviral strategies. This integrative framework positions PTMs as not merely bystanders but as central modulators of viral fitness and host vulnerability, offering novel avenues for therapeutic intervention against SARS-CoV-2 and future pandemic threats.

## 1 Introduction

The COVID-19 pandemic has highlighted SARS-CoV-2’s ability to hijack host cell machinery with remarkable precision (Pham T. X. et al., 2023; Khan and Islam, 2020; Amahong et al., 2025; Yuan et al., 2021; Jackson et al., 2022; Cheng et al., 2022; Mishra et al., 2021). While acute respiratory presentation may predominate in the clinic, the illness tends to transgress the lung (Tsatsakis et al., 2020), manifesting in cardiovascular (Hendren and Ammirati, 2025), neurological (Pang et al., 2024), and immunologic effects like persistent syndromes that have come to be referred to as long COVID (Makhluf et al., 2024). In addition, the repeated emergence of immune-evasive mutants has tested both vaccine-elicited and natural immunity, with far-reaching implications for understanding the molecular determinants of this plasticity. In addition to its genomic plasticity, the virus employs various strategies to manipulate the host environment and evade immune detection (Fan H. et al., 2024; Cao et al., 2025; Massey, 2021; Baggen et al., 2021; Singh et al., 2020). Among these, PTMs have proved to be critical molecular switches that reprogram host signaling pathways (Pellegrina et al., 2022; Guan et al., 2023; Fung et al., 2022), modulate viral protein function (Li X. et al., 2025; Zhao et al., 2024), and reset immune responses (Li X. et al., 2025; Zhou et al., 2017). Even as their roles in viral infection are increasingly appreciated, the scope and functional diversity of PTMs in SARS-CoV-2 infection remain underappreciated.

Post-translational modifications such as phosphorylation, ubiquitination, SUMOylation, glycosylation, and acetylation, among others, dynamically reorganize the structural and functional topology of virus and host proteins (Cheng et al., 2022; Li X. et al., 2025; Fan et al., 2025; Wu et al., 2025; Sun et al., 2024; Shehata et al., 2025; Daly et al., 2023; Kumar et al., 2020; Xue et al., 2022). Apart from regulating protein localization, stability, and interaction affinity (Zhong et al., 2023; Han Y. et al., 2025; Zacharias et al., 2021), these PTMs also serve as mechanisms for rapid responses in antiviral immunity (Zhou et al., 2017; Song et al., 2021). PTMs regulate essential events in the viral life cycle in SARS-CoV-2: they regulate spike glycoprotein conformational dynamics and receptor binding (Huang C. et al., 2021; Oh et al., 2023), regulate nucleocapsid phase separation and RNA binding (Wu et al., 2022), and hijack host kinase and ubiquitin ligase signaling to repress interferon signaling and autophagy (Khatun et al., 2023; Yuan and Ding, 2022; Zhao et al., 2023). In addition, emerging evidence suggests functional crosstalk between PTMs that facilitates synergistic or antagonistic regulation of host defense and commonly shows variant-specific effects.

Here, we present a mechanistic and integrative summary of existing information on PTMs in the context of SARS-CoV-2 infection. We describe how each PTM contributes to viral replication, immune evasion, and tissue tropism, and how they converge at regulatory control sites. By synthesizing information from proteomics, structural biology, and immunology, we try to build a PTM-focused framework of understanding COVID-19 pathogenesis and host-directed antiviral therapies.

## 2 Overview of PTMs: diversity, mechanisms, and relevance to viral infection

PTMs are chemical modifications of amino acid side chains that give rise to a large number of proteins, roughly three times the total number of genes encoded in the human genome (Zhong et al., 2023; Ramazi and Zahiri, 2021; Huang et al., 2014; Mann and Jensen, 2003; Chuh et al., 2016). These chemical changes include phosphorylation (Zhao et al., 2024), S-nitrosylation (Cox et al., 2025), methylation (Khlifa et al., 2025), acetylation (Chen and Zhang, 2024), glycosylation (Feng et al., 2022), O-GlcNAcylation (Dong et al., 2025), ubiquitination (Sparrer et al., 2023), palmitoylation (Han Z. et al., 2025), and other modifications of proteins.

Mechanistically, PTMs function as rapid and reversible molecular switches that fine-tune protein conformation and activity in response to intra- and extracellular cues. Phosphorylation, for instance, modulates enzymatic activity and signaling cascades by adding phosphate groups via kinases and removing them via phosphatases (Ardito et al., 2017; Kinoshita-Kikuta et al., 2024). Ubiquitination, in contrast, often targets proteins for proteasomal degradation, although it can also regulate non-degradative processes such as endocytosis and DNA repair depending on chain topology (Liao et al., 2024; Varadan et al., 2002). SUMOylation, which is a type of post-translational modification that involves covalent conjugation of small ubiquitin-like modifier (SUMO) proteins to target substrates, regulates various critical molecular and cellular processes, including transcription, the cell cycle, cell signaling, and DNA synthesis and repair (Huang et al., 2024; Filippopoulou et al., 2020), while glycosylation and methylation modify protein folding, immune recognition, and epigenetic architecture (Horvat et al., 2011; He et al., 2024; Štambuk et al., 2021). ISGylation, an interferon-induced PTM, modulates antiviral immunity not only through direct conjugation to host and viral proteins, but also by acting as a secreted cytokine-like signal in immune crosstalk (Liu G. et al., 2021; Perng and Lenschow, 2018; Miller et al., 2024).

The functional impact of PTMs extends broadly to viral pathogens, including SARS-CoV-2. This virus has evolved sophisticated mechanisms to exploit, rewire, or evade host PTM systems to optimize its replication, assembly, and immune evasion pathways (Cheng et al., 2022; Rhamadianti et al., 2024; Cao, 2021). Also, SARS-CoV-2 proteins such as ORF6 and ORF10 hijack or degrade host ubiquitin ligases to suppress interferon signaling and disrupt essential cellular functions (Khatun et al., 2023; Yuan and Ding, 2022). In the following sections, we provide a detailed, modification-specific analysis of how individual PTMs, including phosphorylation, ubiquitination, SUMOylation, glycosylation, acetylation, palmitoylation, ADP-ribosylation, succinylation, ISGylation, and neddylation, contribute to the pathogenesis of SARS-CoV-2.

### 2.1 Phosphorylation: the dynamic regulator of viral protein function

#### 2.1.1 Functional implications of nucleocapsid phosphorylation

Phosphorylation of the SARS-CoV-2 nucleocapsid (N) protein functions not merely as a post-translational modification, but as a dynamic molecular code that orchestrates structural conformation, RNA affinity, and condensate behavior, ultimately dictating the switch between transcriptionally active and packaging-competent viral states. Rather than acting in isolation, these phosphorylation events interact across protein domains and time points in infection to create functionally distinct states. At the domain level, S51 phosphorylation of the N-terminal RNA-binding domain directly represses RNA binding while increasing phase separation, suggesting a dual function in promoting stress granule-like condensates and relieving inhibitory interactions with host components (Wu et al., 2022). Yet, whether virus-induced granule-like phase separation promotes or represses viral replication remains a major unknown, demonstrating that phase behavior function is context-dependent rather than structural. Botova et al. (2024) take this further by showing that hyperphosphorylation across the serine/arginine-rich (SR) domain, catalyzed by SRPK1, CK1, and GSK3, abolishes RNA binding entirely through an intramolecular switch in which the phosphorylated SR domain occupies the RNA interface. Interestingly, this is not observed when phosphorylation is catalyzed by protein kinase A, demonstrating the specificity with which recruitment of the host kinase contributes to viral protein topology. These results indicate that, aside from the event, the pattern and origin of phosphorylation are also relevant for establishing functional effects. This offers a conceptual connection between structural state and signaling pathway.

The functional significance of this modulation is deepened by Stuwe et al. (2024), who reveal that SR phosphorylation exerts long-range effects by disrupting a Leu-rich helix required for self-association, located 30 residues away. This allosteric destabilization uncouples RNA binding from phase behavior, a finding that challenges the earlier assumption of their strict interdependence (Stuwe et al., 2024). In this light, phosphorylation emerges as a multi-potential regulator that fine-tunes electrostatics, multimerization, and spatial position in an integrated manner. This argument is further substantiated by Loonen et al. (2025), who used molecular dynamics simulations to show that phosphorylation enhances structural dynamism but reduces the affinity of some contacts with 5′ UTR elements. This would imply that dynamic fine-tuning over absolute on/off regulation is at the core of the N function. Whereas past research highlights binary transitions (Wu et al., 2022; Rouzina et al., 2025), the present investigation suggests a model of graded functional plasticity, a notion underpinned by viral requirements for strict temporal regulation of assembly and replication (Loonen et al., 2025). Bridging structure and virological function, Rouzina et al. (2025) employ phosphomimetic mutants to show that phosphorylation weakens genome compaction capacity while preserving chaperone-like activity, thus promoting subgenomic RNA synthesis. This functional switch, reversible, subtle, and phosphorylation-driven, provides a biochemical basis for the dual roles of the N protein across infection stages. Unlike other studies (Wu et al., 2022; Botova et al., 2024), this work integrates physical and functional assays, offering the most direct evidence that phosphorylation transitions N from a genome-packaging unit to a transcription-facilitating scaffold (Rouzina et al., 2025).

**Figure 1:**
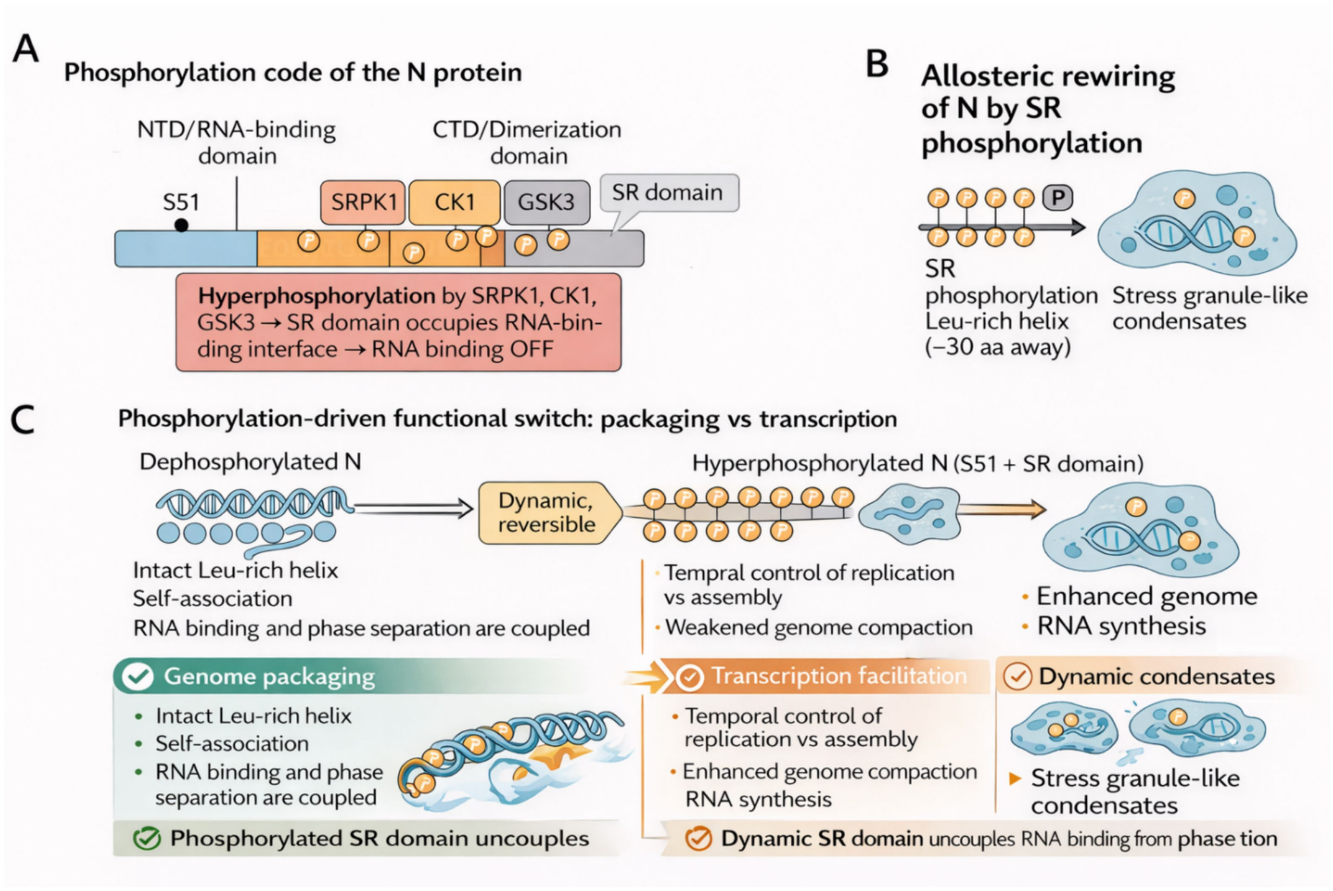
Phosphorylation orchestrates a dynamic regulatory code that controls SARS-CoV-2N protein structure and function. **(A)** Site-specific phosphorylation at S51 and hyperphosphorylation of the SR domain by SRPK1, CK1, and GSK3 remodel the RNA-binding interface, switching the N protein from a high-affinity RNA-bound state to an RNA-disengaged conformation. The SR domain’s phosphorylation is shown to prevent RNA binding by altering its interaction interface. **(B)** Phosphorylation of the SR domain induces long-range allosteric destabilization of the Leu-rich helix (~30 aa away), reducing N self-association and partially uncoupling phase-separation behavior from RNA interactions. The blue blobs represent stress granule-like condensates, formed as a result of the SR domain’s phosphorylation. **(C)** These coordinated modifications generate a reversible functional switch: dephosphorylated N forms compact, packaging-competent ribonucleoprotein structures, whereas hyperphosphorylated N promotes dynamic condensates, weakened genome compaction, and enhanced subgenomic RNA synthesis. The transition between these states illustrates the protein’s dual role in viral replication and transcription.

Together, these studies establish a unifying model in which phosphorylation of the SARS-CoV-2 nucleocapsid protein encodes a functional bifurcation: hyperphosphorylated N facilitates transcriptional activity, dynamic interaction with host factors, and liquid-like phase separation; dephosphorylated N reverts to a stable, high-affinity RNA-binding form optimized for genome packaging. This graded regulation aligns with the virus’s need for precise temporal control over the balance between replication/transcription and genome packaging during distinct phases of the infection cycle, with hyperphosphorylation predominating early to support replication and dephosphorylation facilitating compact assembly in later stages (Carlson et al., 2022; Lu et al., 2021). This bidirectional control is achieved through a complex choreography of site-specific modifications, long-range intramolecular interactions, and kinase-specific signatures. Interestingly, this post-translational code could create a new therapeutic window; pharmacologic blocking of SR phosphorylation pathways could specifically break viral replication cycles without compromising the viability of the host cell. Interestingly, this post-translational code could create a new therapeutic window; pharmacologic blocking of SR phosphorylation pathways could specifically break viral replication cycles without compromising the viability of the host cell. For example, several GSK-3 inhibitors (a key kinase in the SR-domain phosphorylation cascade) have demonstrated potent antiviral activity against SARS-CoV-2 in cell culture models, with high proportions showing low cytotoxicity to host cells (Shapira et al., 2022; Liu X. et al., 2021; Yaron et al., 2022). These multiscale effects, from S51-mediated repression of RNA binding to SR-domain hyperphosphorylation, long-range allostery, and the resulting packaging-to-transcription switch, are integrated in a unified mechanistic framework (Figure 1).

#### 2.1.2 Viral strategy: phosphorylation of accessory and non-structural proteins

Phosphorylation-based modulation of host signaling is a central feature of SARS-CoV-2’s immune evasion strategy. While much attention has focused on structural proteins, a growing body of evidence reveals that accessory and non-structural proteins (Nsps) of SARS-CoV-2 actively exploit, alter, or evade phosphorylation-dependent pathways to fine-tune host antiviral responses. The mechanisms revealed are heterogeneous, ranging from preventing host kinase access to outright interference with phosphorylation cascades, and emphasize the viral capability to usurp host cellular rationality for its replicative advantage. One of the clearest examples of phosphorylation-regulated subversion comes from Orf9b, a small accessory protein that targets the mitochondrial translocase TOM70. Brandherm et al. (2021) demonstrated that Orf9b binding to TOM70 blocks Hsp90 recruitment, thereby attenuating type I interferon responses. Crucially, phosphorylation at serine 53 impairs this interaction, as evidenced by a phosphomimetic mutant (Orf9b S53E) that exhibits markedly diminished binding to TOM70 (Brandherm et al., 2021). This suggests that phosphorylation acts as a negative regulator of immune suppression. Compared to other viral proteins that enhance suppression via phosphorylation, such as NSP13 (Fung et al., 2022), Orf9b’s dependence on the unphosphorylated state introduces a regulatory inversion: phosphorylation disarms rather than arms the viral protein.

NSP13, a helicase, employs a distinct mechanism to dampen interferon signaling by blocking STAT1 phosphorylation. Fung et al. (2022) showed that NSP13 physically blocks JAK1 from phosphorylating STAT1 after binding to STAT1, which suppresses downstream transcriptional activation. Interestingly, both the helicase and NTPase functions of NSP13 were required for this, indicating an interconnection between its traditional enzymatic functions and immune suppression (Fung et al., 2022). In contrast to Orf9b (Brandherm et al., 2021), which targets a chaperone–receptor interface, NSP13 performs pseudo-substrate host-substrate shielding by directly inhibiting kinase activity (Fung et al., 2022). This function is analogous to viral decoys for other viruses and signifies that the virus does not target phosphorylation but is an Achilles’ heel within the host that the virus actively hides.

Building on this paradigm, Caobi et al. (2025) demonstrated that NSP15, an endoribonuclease, increases virulence by inhibiting viral double-stranded RNA (dsRNA) accumulation, thereby preventing phosphorylation-mediated innate immune signaling. NSP15 itself does not directly alter phosphorylation, but its activity inhibits the induction of phosphorylation cascades (e.g., IRF3, TBK1) before these key checkpoints (Caobi et al., 2025). Unlike the direct blockade approaches of Orf9b (Brandherm et al., 2021) and NSP13 (Fung et al., 2022), NSP15 (Caobi et al., 2025) represses immune activation by inhibiting the formation of pathogen-associated molecular patterns (PAMPs) that would otherwise trigger kinase cascades. This proves a multi-layered viral strategy: not everything that is inhibited is done so by a physical block; some are inhibited by substrate unavailability to deny the immune system activating signals.

**Figure 2:**
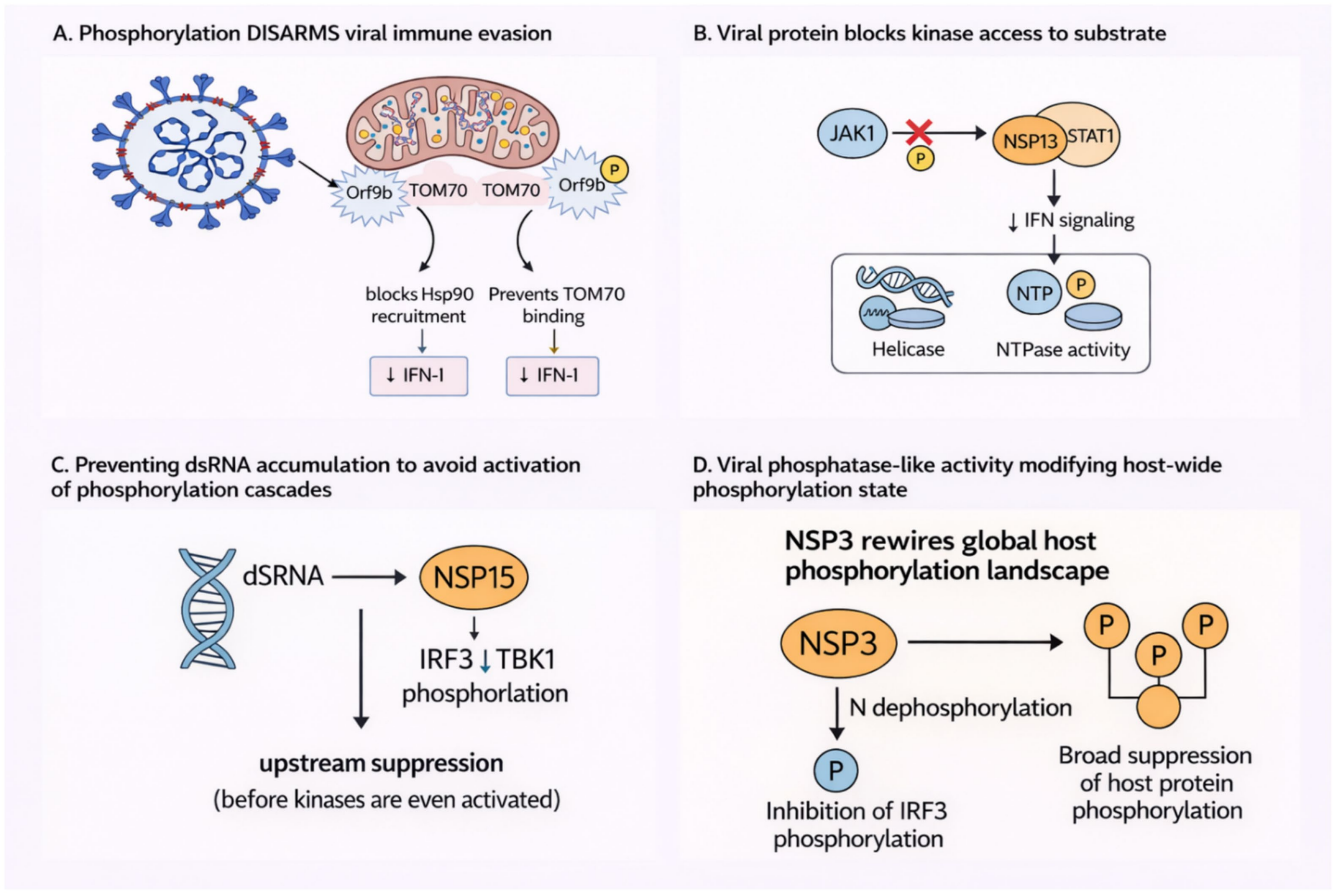
Phosphorylation-dependent antiviral evasion mechanisms used by SARS-CoV-2 accessory and non-structural proteins. **(A)** Phosphorylation of Orf9b at S53 disrupts its interaction with TOM70, abolishing Hsp90 blockade and relieving suppression of type I interferon signaling. **(B)** NSP13 binds STAT1 and prevents JAK1-mediated phosphorylation, suppressing IFN signaling in a helicase/NTPase-dependent manner. **(C)** NSP15 removes viral dsRNA, blocking upstream activation of IRF3 and TBK1 phosphorylation cascades. **(D)** NSP3 acts as a phosphatase-like regulator, promoting N dephosphorylation, inhibiting IRF3 phosphorylation, and broadly reducing host protein phosphorylation to reshape the cellular antiviral landscape.

The most systemic disruption arises from NSP3, which acts as a global-level phosphorylation modulator. Yang et al. (2025) found that NSP3 interacts with the N protein and dephosphorylates it. In addition, NSP3 generally inhibits IRF3 phosphorylation and host protein phosphorylation in a dose-dependent manner. These findings indicate that NSP3 behaves as a viral phosphatase or phospho-docking regulator, rewiring the host phosphorylation environment to support viral replication (Yang et al., 2025). A consolidated overview of these phosphorylation-dependent viral strategies, including Orf9b, NSP13, and NSP3, is shown in Figure 2. The action here is not restricted to a single pathway or target but rather elicits a change in the host cell’s overall signaling tone. In contrast to NSP13 (Fung et al., 2022), which acts at a single immune node, NSP3 introduces network-level rewiring, potentially affecting multiple lines of host defense simultaneously (Yang et al., 2025).

Together, these studies reveal a multifaceted phosphorylation-targeting strategy used by SARS-CoV-2 to escape host immunity. Certain proteins (e.g., Orf9b) use their own phosphorylation states to control interaction fidelity with host receptors, while others (e.g., NSP13, NSP3) directly prevent or reverse host phosphorylation events. This functional diversity highlights phosphorylation pathways as central sites of host-virus conflict. These revelations reinforce host kinase-phosphatase pathways as potential therapeutic targets capable of silencing multiple viral effectors at once.

#### 2.1.3 Host–virus interplay: cellular kinases and functional modulation

Viruses rely extensively on the host’s kinase repertoire to take advantage of intracellular signaling and post-translational control. SARS-CoV-2 is a prime example of such dependency by hijacking cellular kinases by coercion to modulate viral protein activity, reorganize host physiology, and dampen immune function. Instead of tapping into a single pathway, the virus shifts the phospho-signaling network in a biased manner, often including kinases that are otherwise critical for cell cycle, inflammation, or stress pathways. A seminal phosphoproteomics study by Bouhaddou et al. (2020) provided a global view of this rewiring. SARS-CoV-2 infection triggered activation of kinases, including CK2 and p38 MAPK, but also suppressed mitotic regulators, resulting in cytoskeletal remodeling and cell cycle arrest (Bouhaddou et al., 2020). This kinase activation supported the development of CK2-rich filopodia containing viral particles, potentially increasing cell-to-cell transmission. Of particular interest, chemical inhibition of p38, CK2, and CDKs showed antiviral activity, such that these kinases are not silent passengers but dynamic facilitators of viral replication (Bouhaddou et al., 2020). This understanding paved the way for follow-up targeted studies. Upon this foundation, Yaron et al. (2022) described an orchestrated phosphorylation cascade involving SRPK1/2, GSK3, and CK1, which sequentially phosphorylate the N protein. SRPK1/2 inhibition or knockout arrested N phosphorylation and viral replication. These results differ from Bouhaddou’s larger signaling map in that they exhibit causal kinase–substrate–function relationships (Yaron et al., 2022). Notably, conservation of these phosphosites across coronaviruses highlights a common vulnerability that pan-coronaviral therapeutics can target.

Concurrently, in an orthogonal but complementary manner, Guo et al. (2025) demonstrated that SARS-CoV-2 causes HSPA9 to be phosphorylated at Ser627. This modification stimulates mitochondrial biogenesis and suppresses the production of proinflammatory cytokines, such as IL-6 and IL-8. This diversion indicates that the virus can hijack host stress kinases to increase bioenergetic support and suppress early immune responses. MAPKAPK2 was identified as a potential upstream kinase that links viral replication requirements and immune evasion (Guo et al., 2025). In contrast to N phosphorylation studies (Liu X. et al., 2021; Yaron et al., 2022), this work reveals how SARS-CoV-2 repurposes host stress kinase networks (e.g., via MAPKAPK2-mediated HSPA9 phosphorylation) to simultaneously enhance bioenergetic support and suppress early immune responses, highlighting a potential axis of host kinase adaptation to viral needs (Guo et al., 2025). At the systems level, Shaji et al. (2025) undertook an integrative phosphomotif and interactome mapping of 33 SARS-CoV-2 proteins. They recognized 49 host kinases at 639 phosphosites and revealed MAP2K1 as a high-confidence kinase binding conserved N and Orf9b sites. Compared to SRPK1 (Yaron et al., 2022), MAP2K1 displayed stronger binding affinity in docking studies (Shaji et al., 2025). This work is distinct in offering a computational–structural prioritization pipeline that enables rational repurposing of kinase inhibitors for antiviral use. A novel mechanism of kinase utilization is explained by Teratake et al. (2025), who demonstrated that ALK1-mediated SMAD5 phosphorylation through spike–ACE2 interaction increases inflammatory gene expression. Notably, this activity was SARS-CoV-2 virus-specific and not present in the less virulent NL63 coronavirus (Teratake et al., 2025). An indication that receptor binding is not uniform is that SARS-CoV-2 uses ACE2 as a signaling platform, hijacking BMP receptor pathways to enhance immunopathology.

Collectively, these observations demonstrate a multilevel hijacking of host kinases by SARS-CoV-2: from cell reprogramming (CK2, MAPK) and site-specific phosphoregulation (SRPK1, MAP2K1) to cytokine regulation (MAPKAPK2) and amplification of proinflammatory signaling (ALK1). Whereas some kinases have prominent roles in viral replication (e.g., SRPK1/2), others confer contextual advantage by reprogramming cell cycle, metabolism, or immune tone. The convergence of global and targeted research shows that host kinases are not incidental bystanders but are instead positive drivers of SARS-CoV-2 pathogenesis and, therefore, high-priority targets for host-targeting therapies.

### 2.2 Ubiquitination—modulating the balance between viral persistence and host immunity

#### 2.2.1 Hijacking host E3 ligases: subversion of cellular machinery

Viruses have developed advanced ways of hijacking the host ubiquitin-proteasome system (UPS), a cellular regulatory complex at the center of the cellular organism, to their advantage. For SARS-CoV-2, this hijacking is complex and selectively advanced to downscale antiviral immunity and re-engineer host cell biology. Several recent works examine the specific manipulation of host E3 ligases by the virus, either by exploiting them to remove antiviral factors or by bypassing their regulatory activity. In one such pioneering work, Wang et al. (2021) found that the host E3 ligase Skp2, already described as an oncoprotein, is involved in mediating the degradation of the ACE2 receptor after benzo(a)pyrene (BaP) treatment, a tobacco carcinogen. This counterintuitively reduces cellular permissiveness to viral infection despite heightened ACE2 mRNA expression. Interestingly, Skp2 was induced AhR-dependently, providing a link between environmental exposure and the regulation of viral receptor levels (Wang et al., 2021). Although not viral hijacking, this illustrates how environmental factors can redirect E3 ligase activity, conferring apparent resistance to infection. But due to the oncogenic role of Skp2, writers rightfully advise against an interpretation of tobacco smoking’s protective role, citing the subtle interaction between carcinogens, host proteostasis, and viral pathogenesis. Khatun et al. (2023), however, provide an eminently textbook example of viral subversion: the SARS-CoV-2 ORF6 protein degrades the host antiviral E3 ligase TRIM25. TRIM25 is instrumental in promoting K63-linked ubiquitination of the viral RNA sensor RIG-I to activate type I IFN signaling. Silencing by TRIM25 degradation adds another level to the already known capability of ORF6 to inhibit IRF3 and STAT1 nuclear import, solidifying its position as a key antagonist of host antiviral defense mechanisms (Khatun et al., 2023). This mechanism adds another layer to ORF6’s known ability to inhibit nuclear import of IRF3 and STAT1, reinforcing its designation as a central antagonist of host antiviral defenses (Khatun et al., 2023). Significantly, this work identifies a clear structure–function interaction between ORF6 and its host target, in contrast to the more indirectly characterized modulation reported in Wang et al. (2021).

A recent review by Pellman et al. (2024) comprehensively discusses the opposing roles of human E3 ubiquitin ligases as both effectors and targets in SARS-CoV-2 infection, highlighting how the virus exploits or evades these ligases to suppress antiviral defenses while host ligases can attenuate infection. Additionally, Zhang L. et al. (2024) identified that the CRL4B E3 ligase complex, recruited by PRPF19, targets SARS-CoV-2 ORF6 for ubiquitin-dependent proteasomal degradation, thereby counteracting ORF6-mediated interferon inhibition and restricting viral replication.

**Figure 3:**
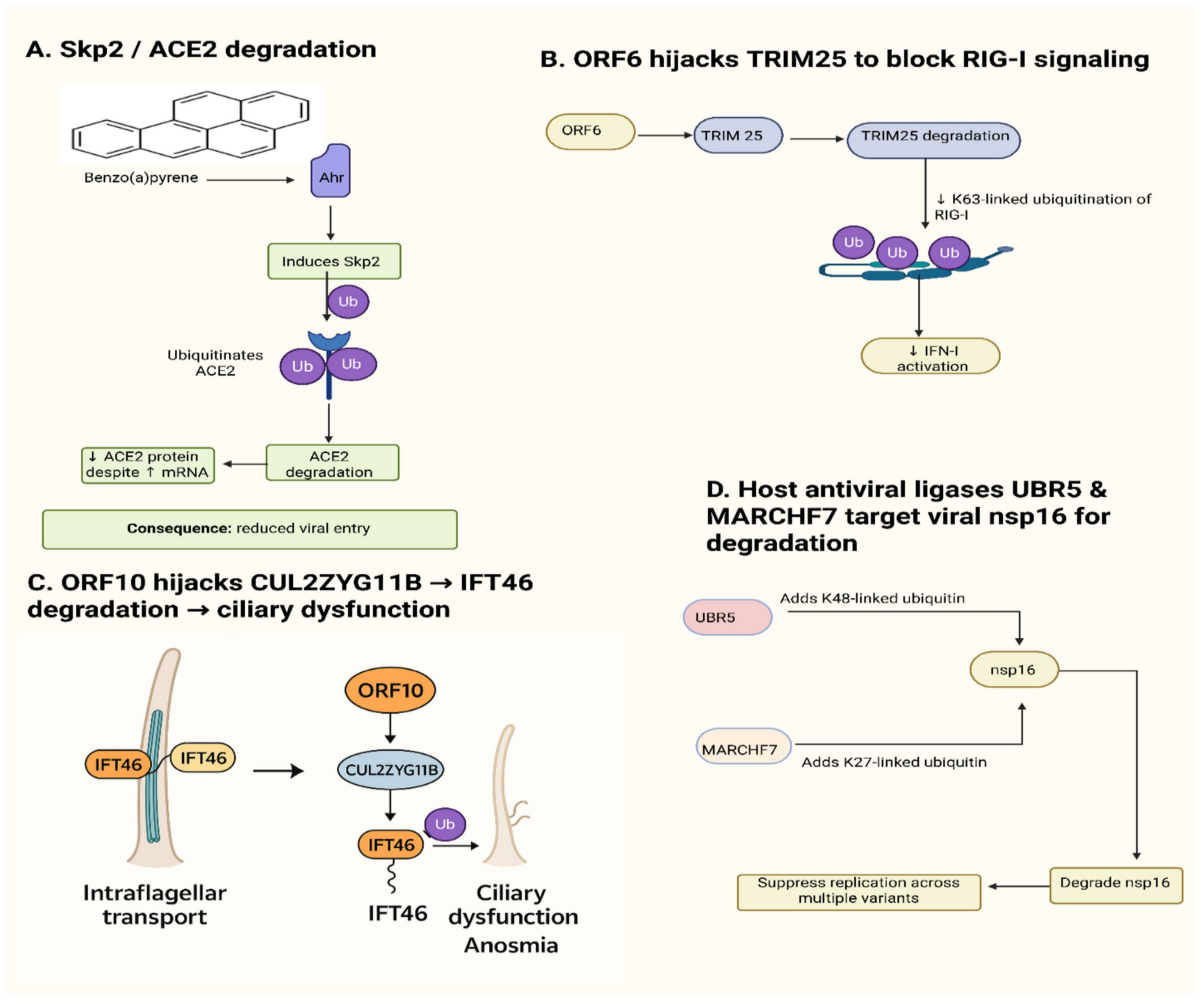
Viral subversion and host deployment of E3 ubiquitin ligases in SARS-CoV-2 infection. **(A)** Environmental activation of AhR drives Skp2 upregulation, promoting ACE2 ubiquitination and degradation despite elevated mRNA levels. **(B)** SARS-CoV-2 ORF6 targets the antiviral ligase TRIM25 for degradation, suppressing K63-linked ubiquitination of RIG-I and downstream IFN-I signaling. **(C)** ORF10 co-opts the CUL2ZYG11B complex to ubiquitinate and degrade IFT46, impairing intraflagellar transport and contributing to ciliary dysfunction. **(D)** In contrast, host ligases UBR5 and MARCHF7 exert antiviral activity by installing K48- and K27-linked ubiquitin chains on the viral methyltransferase nsp16, promoting its degradation and restricting replication across variants.

Moving further into proteomic complexity, Zhu K. et al. (2024) demonstrate that ORF10, a less-well-characterized SARS-CoV-2 protein, interacts with the substrate adaptor CUL2^ZYG11B^, a member of the cullin-RING E3 ligase complex. The interaction is similar to a Gly/N-degron motif and is permissive for degradation of IFT46, a subunit of the intraflagellar transport apparatus essential for ciliary function (Zhu K. et al., 2024). Loss of ciliary integrity is one of the most distinguishing aspects of COVID-19 pathogenesis, perhaps tying this ORF10-targeted hijacking to clinical presentations such as anosmia and compromised mucociliary clearance (Zhu K. et al., 2024). However, these findings remain controversial, as Mena et al. (2021) reported that, while the ORF10–ZYG11B interaction is confirmed, depletion of ZYG11B (or its paralog ZER1) does not impair SARS-CoV-2 replication *in vitro*, and there is no evidence that ORF10 functionally hijacks or modulates CRL2^ZYG11B^ activity. Notably, the structural definition of the ORF10–CUL2^ZYG11B^ complex not only provides insight into this mechanism but also represents a promising area of research for PROTAC-guided drug design, leveraging viral mimicry to repurpose host ubiquitination mechanisms therapeutically—pending resolution of these conflicting functional data. This is in contrast to a study by Tian et al. (2025), which paints a different picture of how the host E3 ligases UBR5 and MARCHF7 act as anti-viral brakes by targeting the SARS-CoV-2 methyltransferase nsp16 for degradation. UBR5 recruits K48-linked chains, which are generally used in canonical proteasomal degradation, whereas MARCHF7 initiates K27-linked chains, the mechanism of action of which remains poorly understood but has seemingly convergent antiviral effects (Tian et al., 2025). Both the ligases inhibited SARS-CoV-2 replication *in vivo* and *in vitro* in several viral mutants (Tian et al., 2025). This places the E3 ligases not only as virus-hijacked tools but also as host-intrinsic inhibitors of infection, underscoring their double-edged roles depending on the context of interaction. These mechanistically distinct modes of E3-ligase manipulation, from Skp2- and TRIM25-directed subversion to CUL2ZYG11B hijacking and nsp16 restriction, are integrated in the Figure 3.

Cumulatively, these findings provide a rich portrait of host–virus interactions focused on E3 ligases. From viral proteins that recruit host E3 ligases to ubiquitinate and degrade host immune actors (e.g., TRIM25 through ORF6 recruitment) or rewire cellular networks (e.g., IFT46 through ORF10-mediated activation of CUL2^ZYG11B^), to host factors that block infection (e.g., UBR5 targeting nsp16), the ubiquitination axis is a war zone as well as a therapeutic target. Structural mimicry of ORF10 and functional promiscuity of the E3 ligases highlight the complexity of the viral hijacking mechanism. In particular, although degradation of ACE2 by Skp2 in smokers superficially appears to confer a protective response, it also shows that carcinogenic reprogramming of the E3 ligases may cross-talk with viral biology and raises significant issues about the long-term effects of such modulation.

#### 2.2.2 Ubiquitin-mediated control of viral protein stability and replication

Ubiquitination is also a central regulatory event in SARS-CoV-2 infection, impacting viral protein stability, viral replication kinetics, and host immune signaling. In various recent research studies, degradative and non-degradative ubiquitin signals were each implicated in preventing viral progression or inducing viral pathogenesis. At the level of viral enzymatic machinery, PLpro, the SARS-CoV-2 papain-like protease, is a central nexus regulating both polyprotein cleavage and immune evasion by deubiquitination. Engineered variants of ubiquitin (UbVs) in a paper by van Vliet et al. (2022) were found to bind at a distal position relative to the catalytic site of PLpro but allosterically inhibit its cleavage activity. The inhibitor inhibited virus replication in cells by up to 5 logs and is an illustration of the capability of non-competitive Ub-mimetics to target viral replication and immune evasion simultaneously (van Vliet et al., 2022). Concurrently, two host E3 ligases, Parkin and ZBTB25, were independently found to destabilize Mpro, the primary viral protease. ZBTB25 specifically binds to Mpro and ubiquitinates it at Lys100, resulting in proteasomal degradation and impaired infection (Lear et al., 2023). At the same time, Parkin induced during mitophagy by PINK1 targets Mpro and inhibits viral replication (Zhou L. et al., 2024). Interestingly, SARS-CoV-2 suppresses Parkin expression in lung tissue, an evolutionary countermeasure to suppress this antiviral pathway (Zhou L. et al., 2024). These results demonstrate that different ligases can target the same viral target (Mpro) through distinct upstream stimuli and cell contexts.

Recent studies further highlight host E3 ligases targeting key viral replication proteins. Fan L. et al. (2024) showed that TRIM22, an interferon-stimulated E3 ligase, promotes K48-linked ubiquitination and proteasomal degradation of NSP8 at Lys97, thereby restricting SARS-CoV-2 replication. Similarly, Zhou et al. (2024b) identified FBXO22 as an E3 ligase that catalyzes K48-linked polyubiquitination of NSP5 (the main protease) at Lys5 and Lys90, leading to its degradation and inhibition of viral replication.

In contrast, modulation of the membrane (M) protein illustrates a dual regulatory landscape. RNF5 ubiquitinates M at Lys15, enhancing its interaction with envelope (E) protein and promoting virion assembly and release via the autophagy pathway (Yuan and Ding, 2022). This function is opposed by the deubiquitinase PSMD14, which limits M–E interaction and budding (Yuan and Ding, 2022). Conversely, TRIM7 ubiquitinates M at a nearby site (K14), but in this context acts to prevent apoptosis and restrict replication (Gonzalez-Orozco et al., 2024). Improved viral burden and lung pathology in TRIM7-deficient mice correlate with site-specific ubiquitination of nearby lysines (K14 vs. K15), with disparate consequences for host survival vs. viral release (Gonzalez-Orozco et al., 2024). Strikingly, SARS-CoV-2 patient isolates carry mutations at these residues, implicating evolutionary selective pressure on this UPS-controlled interface. In addition to proteolytic regulation, SARS-CoV-2 exploits non-degradative ubiquitination to escape host immunity (Nishitsuji et al., 2022). NSP6 and ORF7a induce K63-linked ubiquitination by TRIM13 and RNF121, respectively, which induces TAK1–NEMO complex assembly and strong NF-κB activation (Nishitsuji et al., 2022). Such interactions enable the synthesis of proinflammatory cytokines, characteristic of severe COVID-19, and demonstrate how the virus subverts the UPS to enhance pathogenic inflammation.

Zhou et al. (2024a) described a Cullin 5-based E3 complex involving TOM70 and HSP90α that ubiquitinates ORF9b at K67, promoting its proteasomal degradation and acting as a host antiviral mechanism, while HSP90α stabilizes ORF9b. Hua et al. (2024) revealed that NSP14 recruits the linear ubiquitin assembly complex (LUBAC) via HOIP to undergo linear (M1-linked) ubiquitination, enabling recruitment of NEMO and activation of proinflammatory NF-κB signaling without inducing type I interferon.

Ubiquitin-regulated regulation of SARS-CoV-2 proteins marks an active, multi-faceted battlefield between host and virus. Through proteasomal degradation of viral enzymes (ZBTB25, Parkin), regulation of structural proteins (RNF5, TRIM7), or inflammatory activation of host signaling (TRIM13, RNF121), the ubiquitin-proteasome system is a central regulatory hub. Targeting this system with Ub-mimetic inhibitors, PROTACs, or ligase-specific modulators represents a desirable therapeutic approach to interrupt the viral life cycle at multiple nodes while restoring immune homeostasis. Generally, SARS-CoV-2 tailors ubiquitination, amplifying it mechanistically and spatially to maximize viral packaging, dampening it to avoid immunity, and remodeling it in specific tissues. Appreciation of such versatility forms the basis for designing targeted, context-dependent antiviral interventions.

#### 2.2.3 Ubiquitination in immune regulation: a double-edged sword

Ubiquitination has emerged as a key regulator of antiviral immunity, with the potential to enhance host defense or facilitate virus hijacking for immune evasion. Che et al. (2022) offered a system-level view through consolidating multi-organ transcriptomic profiles in COVID-19 patients. They observed increased ubiquitination—i.e., in lymphoid and immune-enriched tissues with increased alveolar immune infiltration, dampened systemic inflammation, and better prognosis (Che et al., 2022). Stronger ubiquitination signatures were correlated with reduced need for mechanical ventilation and intensive care unit therapy and indicated immune-calibration-mediated protective functions (Che et al., 2022). By focusing on specific molecular mechanisms, Mao et al. (2023) found that TRIM21 is an E3 ligase that ubiquitinates the SARS-CoV-2N protein. N protein proteasomal degradation after TRIM21-catalyzed K48-linked polyubiquitination was identified in several variants from Alpha to Omicron (Mao et al., 2023). This not only suppressed the assembly of the virus but also, most likely, contributed to decreased cytokine-mediated pathology. Contrasting with that of Che et al. (2022), which placed global ubiquitin profiles in the foreground, Mao et al. (2023) demonstrated a direct antiviral function of ubiquitination against a viral structural protein.

**Figure 4:**
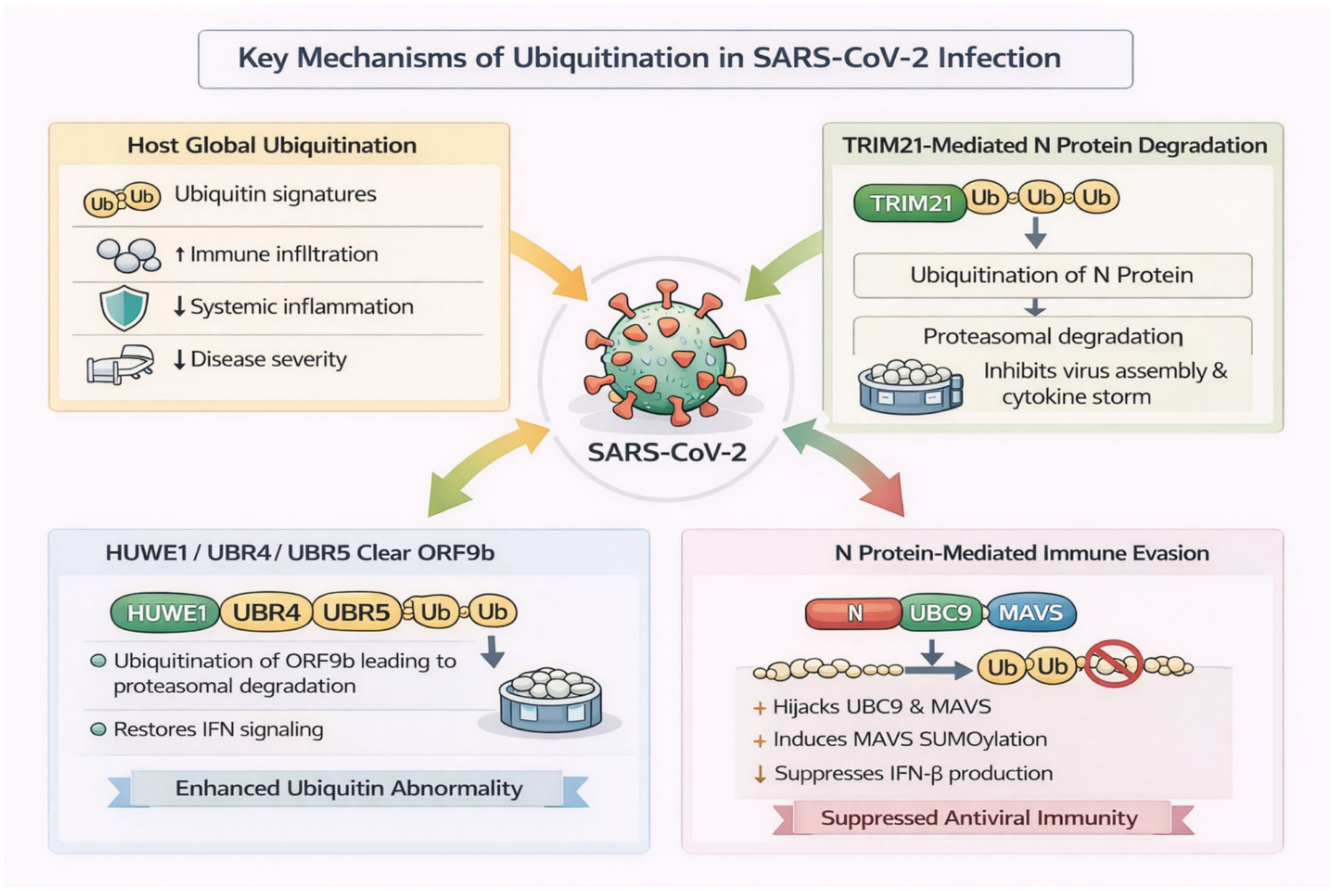
Dual roles of ubiquitination in host antiviral defense and SARS-CoV-2 immune evasion. Heightened global ubiquitination correlates with increased immune infiltration, reduced systemic inflammation, and lower clinical severity in COVID-19. The host ligase TRIM21 catalyzes K48-linked ubiquitination of the viral N protein, promoting its proteasomal degradation and limiting virion assembly and cytokine pathology. HUWE1, UBR4, and UBR5 drive K48-linked degradation of the interferon antagonist ORF9b, restoring IFN-I/III signaling. Conversely, SARS-CoV-2N protein subverts UBC9–MAVS signaling by enhancing MAVS SUMOylation and reducing its ubiquitination, thereby suppressing TBK1/IRF3 activation and IFN-β production.

Yu et al. (2024) identified three additional E3 ligases, HUWE1, UBR4, and UBR5, that individually catalyze ubiquitination and proteasomal degradation of ORF9b, a known SARS-CoV-2 interferon antagonist protein. Their degradation abolished the inhibition of the IFN-I/III pathway, thereby amplifying the antiviral response (Yu et al., 2024). While Mao et al. (2023) targeted structural proteins, Yu et al. (2024) broadened the antiviral function of ubiquitination to immune-modulatory viral proteins. Notably, the two reports Mao et al. (2023) and Yu et al. (2024) agree that host E3 ligases can selectively target viral critical components, activating interferon-dependent immunity through divergent yet complementary mechanisms. But it is bidirectional. Huang et al. (2023) reported a viral countermeasure in which the SARS-CoV-2N protein increases UBC9-MAVS interactions, leading to MAVS SUMOylation. This alteration competitively inhibits its ubiquitination, interferes with downstream phosphorylation cascades of IKKα, TBK1, and IRF3, and ultimately inhibits IFN-β expression (Huang et al., 2023). This is how SARS-CoV-2 appropriates the ubiquitin–SUMO interaction to suppress innate antiviral signaling. The work contrasts starkly with that of Mao et al. (2023): whereas one E3 ligase degrades N for host defense, N appropriates host machinery to safeguard the virus (Huang et al., 2023). Figure 4 integrates these mechanistically divergent roles of ubiquitination, from host-driven antiviral clearance to viral exploitation of SUMO–ubiquitin crosstalk, illustrating its dual function in immune regulation. Guo et al. (2021) demonstrated that SARS-CoV-2 nsp13 hijacks the host deubiquitinase USP13 to deubiquitinate and stabilize itself, thereby suppressing type I IFN production by disrupting TBK1 recruitment to MAVS.

In summary, these studies collectively present ubiquitination as a two-edged sword in immune regulation. Host-driven ubiquitination, whether universal (Zhou L. et al., 2024) or selectively via E3 ligases (Fan L. et al., 2024; Zhou et al., 2024b), enhances viral clearance and immune restoration. In contrast, when hijacked by viral protein (Huang et al., 2023), ubiquitination pathways are redirected to inhibit interferon responses and maintain infection. Therapeutic approaches that enhance productive ubiquitination or prevent its viral co-option may therefore represent viable paths to antiviral therapy.

#### 2.2.4 Expanding horizons: non-canonical and context-specific ubiquitination

Ubiquitination during SARS-CoV-2 infection is no longer a marginal post-translational modification but a highly adaptive process hijacked and controlled by the virus across many cellular contexts. Xu et al. (2022) used a multiomics approach and demonstrated that SARS-CoV-2 hijacks host ubiquitin systems in a specific manner, even in the absence of viral E3 ligases, by enhancing the ubiquitination of structural proteins such as Spike, thereby directly increasing viral infectivity. Their screen also identified four host E3 ligases that regulate infection, with therapeutic promise. Khatun et al. (2023), on the other hand, discovered an immune-suppressive counterpart: SARS-CoV-2 ORF6 recruits TRIM25 for degradation, curtailing K63-linked ubiquitination of RIG-I and inhibiting type I IFN responses. Compared to the viral enhancement of ubiquitination observed in Nishitsuji et al. (2022), this study Khatun et al. (2023) shows deliberate inhibition of immune signaling via targeted E3 ligase degradation, a dichotomy that underscores the virus’s contextual modulation of the ubiquitin system. Liu Z. et al. (2022) reported that ubiquitination of ORF7a prevents its recruitment of BclXL to the ER, thereby attenuating ORF7a-induced ER stress activation and cell apoptosis, allowing the virus to evade host-induced cell death (Table 1).

**Table 1:** Integrated map of SARS-CoV-2 phospho-regulatory mechanisms and computational models predicting their functional outcomes.

Viral protein/host target/computational model	Phosphorylation event/prediction mechanism	Functional outcome/model output	Pathway/host effect/application	References
N protein – S51	S51 phosphorylation represses RNA binding; increases LLPS	Switch RNA-binding ↔ condensate	Regulates transcription–packaging transition	Wu et al. (2022)
N protein – SR domain	SRPK1/CK1/GSK3 hyperphosphorylation blocks RNA binding	Complete inhibition of RNA interaction	Topology controlled by kinase pattern	Botova et al. (2024)
N protein – allosteric helix	SR phosphorylation disrupts helix 30 aa away	Reduced self-association	LLPS uncoupled from RNA binding	Stuwe et al. (2024)
N protein – dynamics	Phosphorylation increases flexibility; reduces UTR5’ affinity	Fine-tuned RNA binding	Dynamic regulatory mechanism	Loonen et al. (2025)
N protein – 6D mutant	Phosphomimetic weakens genome compaction	↑ sgRNA synthesis	Switch to transcriptional scaffold	Rouzina et al. (2025)
Orf9b	S53 phosphorylation disrupts TOM70 binding	Loss of immune antagonism	Restores IFN-I signaling	Brandherm et al. (2021)
NSP13	Blocks JAK1 → STAT1 phosphorylation	IFN suppression	Shutdown of JAK–STAT pathway	Fung et al. (2022)
NSP3	Dephosphorylates N + IRF3	Global antiviral suppression	Remodels host phospho-network	Yang et al. (2025)
CK2/p38 MAPK	Global kinase activation	Filopodia + mitotic arrest	Promotes viral spread	Bouhaddou et al. (2020)
SRPK1/2–CK1–GSK3	Sequential phosphorylation of N	Required for viral replication	Key host kinase dependence	Yaron et al. (2022)
HSPA9	Ser627 phosphorylation	↑ mitochondria; ↓ IL-6	Host metabolic rewiring	Guo et al. (2025)
MAP2K1	Strong computational binding to viral motifs	Antiviral kinase target	Kinase–virus interface	Shaji et al. (2025)
SMAD5	Spike–ACE2 → ALK1 phosphorylation	↑ inflammatory genes	BMP pathway activation	Teratake et al. (2025)

In a non-canonical twist, Zhou J. et al. (2024) identified TRIM6-catalyzed K29-linked ubiquitination of the N protein at residues K102, K347, and K361, which enhances its viral RNA-binding function and replication. This non-degradative K29 linkage defies the typical K48/K63 chains and is an operational repurposing of the host ligases for viral benefit (Zhou J. et al., 2024). Notably, while Khatun et al. (2023) demonstrate E3 ligase repression, Zhou J. et al. (2024) demonstrate their viral co-option in two ways, with the same advantage for the virus. Wang Q. et al. (2024) further expanded the spatial aspect by characterizing the ubiquitinome of SARS-CoV-2-infected mouse brain tissue. Broad modification of neuronal proteins and synaptic and cognitive function pathways was identified, demonstrating that ubiquitination plays a role in neuro-COVID pathogenesis (Wang Q. et al., 2024). This spatial rearrangement adds to previous immune-oriented studies and underlines the systemic scale of virus-encoded ubiquitin regulation. To summarize the mechanistic diversity and host–virus interactions mediated by ubiquitination, Table 2 provides an integrated overview of the major ubiquitin-dependent regulatory events identified across SARS-CoV-2 proteins and host factors.

**Table 2:** Overview of SARS-CoV-2–modulated ubiquitination events and their functional consequences across viral and host pathways.

Viral/host factor	Ubiquitination mechanism/event	Functional outcome	Pathway/host effect	References
Skp2 (host E3 ligase)	BaP-induced Skp2 degrades ACE2	Reduces ACE2 protein despite ↑ mRNA	Environmental regulation of viral receptor levels	Wang et al. (2021)
ORF6	Degrades TRIM25	Blocks K63-linked RIG-I ubiquitination	Suppresses type I IFN signaling	Khatun et al. (2023)
ORF10	Interacts with CUL2ZYG11B → degrades IFT46	Loss of ciliary integrity	Links to anosmia + mucociliary dysfunction	Zhu K. et al. (2024)
UBR5/MARCHF7	UBR5 (K48) + MARCHF7 (K27) ubiquitinate nsp16	Degradation of nsp16	Antiviral suppression of replication	Tian et al. (2025)
PLpro	UbVs bind distally → inhibit PLpro allosterically	Blocks cleavage and deubiquitination	Reduces replication by 5 logs	van Vliet et al. (2022)
ZBTB25	Ubiquitinates Mpro at K100	Proteasomal degradation of Mpro	Reduced infection	Lear et al. (2023)
Parkin	Targets Mpro during mitophagy	Degrades Mpro	Antiviral; virus suppresses Parkin	Zhou L. et al. (2024)
RNF5	Ubiquitinates M at K15	Enhances M–E interaction	Promotes virion assembly & release	Yuan and Ding (2022)
PSMD14	Deubiquitinates M	Reduces M–E interaction	Limits budding	Yuan and Ding (2022)
TRIM7	Ubiquitinates M at K14	Restricts replication & prevents apoptosis	Opposes virion release	Gonzalez-Orozco et al. (2024)
NSP6/ORF7a	Induce TRIM13 and RNF121 K63 ubiquitination	TAK1–NEMO activation	NF-κB–mediated cytokine induction	Nishitsuji et al. (2022)
Global ubiquitination (patients)	Elevated ubiquitination signatures	Better prognosis and immune calibration	Reduced ICU/ventilation needs	Che et al. (2022)
TRIM21	K48-linked ubiquitination of N	Proteasomal degradation of N	Antiviral; reduces assembly and cytokine pathology	Mao et al. (2023)
HUWE1/UBR4/UBR5	Ubiquitinate ORF9b	Degradation of ORF9b	Restores IFN-I/III signaling	Yu et al. (2024)
N protein (viral)	Increases UBC9–MAVS SUMOylation → blocks MAVS ubiquitination	Prevents activation	Inhibits IKKα/TBK1/IRF3 → ↓ IFN-β	Huang et al. (2023)
Spike (host-regulated)	Enhanced ubiquitination increases infectivity	Greater viral entry	Host E3 ligase modulation of infection	Xu et al. (2022)
TRIM25 (targeted by ORF6)	Degraded by ORF6	Loss of RIG-I activation	Innate immune suppression	Khatun et al. (2023)
N protein (TRIM6)	K29-linked ubiquitination	Boosts RNA binding & replication	Non-canonical ubiquitin signaling	Zhou J. et al. (2024)
Neuronal proteins	Widespread ubiquitinome remodeling	Synaptic and cognitive dysfunction	Neuro-COVID pathogenesis	Wang Q. et al. (2024)

### 2.3 SUMOylation and viral persistence

#### 2.3.1 SUMOylation of viral proteins: functional implications in nucleocapsid and spike

SUMOylation has also become a context-dependent regulatory mechanism that controls SARS-CoV-2 protein function. Madahar et al. (2023) demonstrated that the N protein is SUMOylated at three lysine residues, K65 being most important in promoting its oligomerization and nuclear localization. These changes enable viral RNA packaging and potential nuclear interactions with host factors, indicating that N protein SUMOylation enables productive virion assembly and may contribute to immune modulation by nuclear reprogramming (Madahar et al., 2023). Wang et al. (2025) provided the most detailed mechanistic analysis of spike SUMOylation. They showed distinct roles of SUMO1 and SUMO2 in spike regulation: SUMO1 promotes spike trimerization and virion release, and SUMO2 promotes spike cleavage and cell-to-cell transmission (Wang et al., 2025). These activities are mediated by conserved SIMs and lysines at positions 129 and 1,269. Surprisingly, they identified a cell-penetrating peptide (cpSIM2) that inhibits the spike-SUMO interaction and effectively suppresses SARS-CoV-2 infection in cell culture and in hACE2-transgenic mice (Wang et al., 2025).

Comparatively, Madahar et al. (2023) concentrate on SUMO-mediated augmentation of viral assembly by N protein, whereas Wang et al. (2025) discuss transmission-linked SUMO modification of spike (Wang et al., 2025). Both highlight the use of SUMO as a viral cofactor and propose, by showing that interference with SUMO-spike interactions is an acceptable therapeutic option. Zargan et al.’s (2024) results, while from SUMO fusion constructs, add to this view by proposing a further protective function against spike aggregation. Overall, SUMOylation differentially regulates SARS-CoV-2 proteins to facilitate nuclear targeting and N self-association, as well as spike trafficking and transmissibility. These activities position SUMOylation as a multivalent regulatory interface with druggable promise across multiple stages of the viral life cycle.

#### 2.3.2 Host regulatory networks: TRIM-mediated SUMOylation and antiviral control

SUMOylation has emerged as a double-edged regulator of viral pathogenesis, in which TRIM family members balance the host’s protection against viral replication. Ren et al. (2024) recognized TRIM28 as an E3 SUMO ligase that is proviral and modifies SARS-CoV-2N at lysine 65, facilitating its oligomerization, RNA binding, and liquid–liquid phase separation (LLPS) properties that suppress innate immune stimulation (Ren et al., 2024). Notably, the R203K mutation creates a new SUMOylation site, thereby enhancing these immunosuppressive activities. Disruption of TRIM28–N interaction by a peptide has efficiently inhibited LLPS and re-established antiviral signaling, making TRIM28 a druggable proviral cofactor (Ren et al., 2024). In a distinct proviral mechanism, the SARS-CoV-2 main protease Nsp5 has been shown to upregulate SUMOylation of the host adaptor protein MAVS, enhancing MAVS stability and triggering hyperactivation of the NF-κB pathway. This results in increased expression of proinflammatory cytokines (e.g., IL-1β, IL-6, TNF-α) and may contribute to the cytokine storm in severe disease (Li et al., 2021).

**Figure 5:**
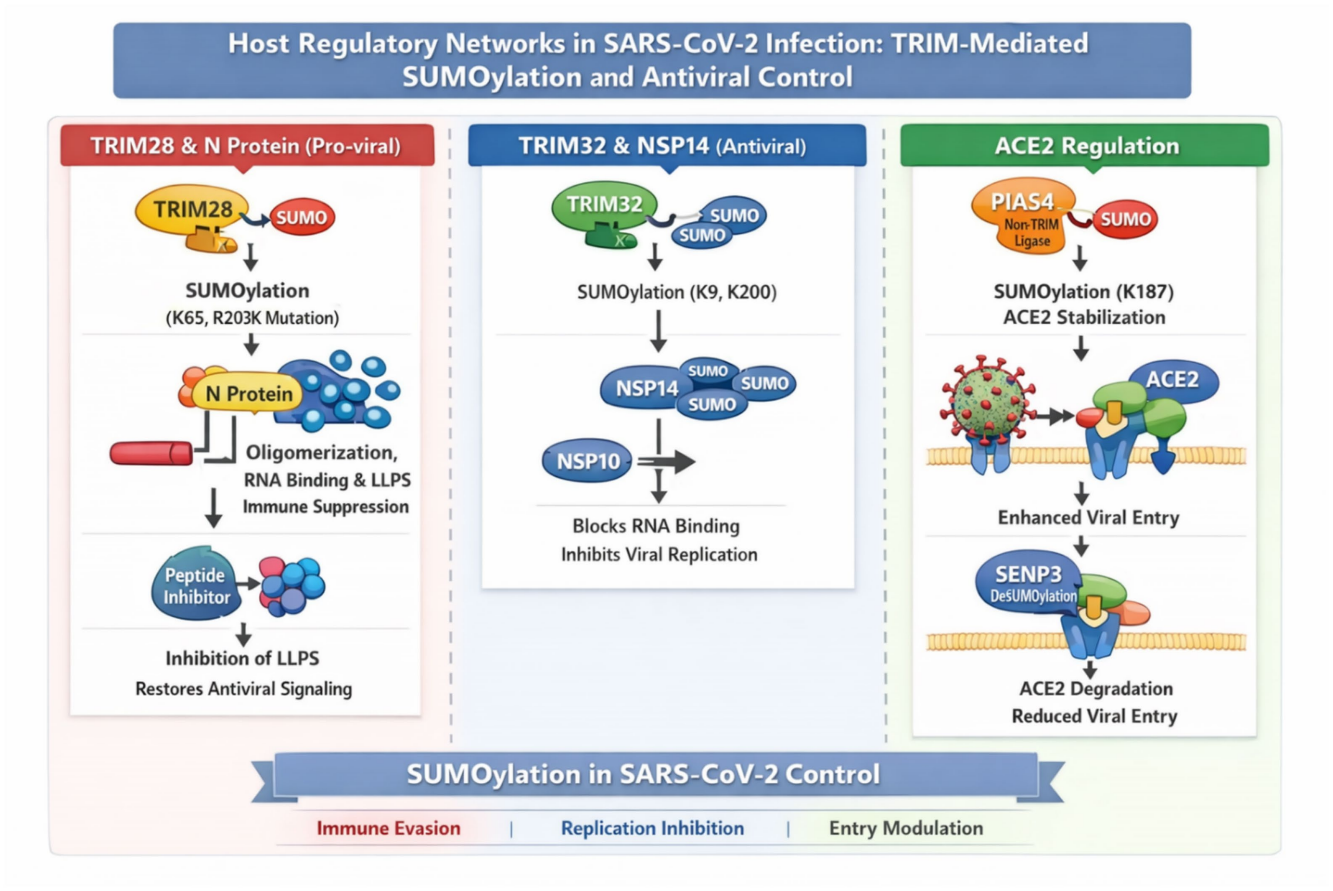
Host regulatory networks in SARS-CoV-2 infection: TRIM-Mediated SUMOylation and antiviral control. This figure illustrates the dual role of SUMOylation in regulating viral pathogenesis and host defense during SARS-CoV-2 infection. The left panel highlights TRIM28 as a proviral factor, promoting N protein oligomerization, RNA binding, and liquid–liquid phase separation (LLPS) through SUMOylation, facilitating immune evasion. The central panel depicts TRIM32, which acts as an antiviral factor, SUMOylating the viral exonuclease NSP14 to block its RNA binding and replication functions. The right panel shows the regulation of the host ACE2 receptor, where PIAS4-mediated SUMOylation stabilizes ACE2, enhancing viral entry, while SENP3-mediated deSUMOylation leads to ACE2 degradation and reduced infection. Collectively, these pathways underscore the functional versatility of SUMOylation in modulating viral replication, immune response, and entry dynamics.

In contrast, Balakrishnan et al. (2025) established that TRIM32 is a host restriction factor. This E3 ligase SUMOylates the viral exonuclease NSP14 at K9 and K200, affecting RNA binding and the recruitment of NSP10, crucial functions for the accuracy of viral replication. TRIM32 was found to inhibit its antiviral activity independently of interferon, suggesting an intrinsic limitation pathway shared by coronaviruses (Balakrishnan et al., 2025). While the structural protein function enhancement by TRIM28’s (Ren et al., 2024) is the reverse of what TRIM32 specifically disables, the enzymatic machinery required for replication (Balakrishnan et al., 2025). Jin et al. (2022) introduced a third dimension of regulation by acting on the host ACE2 receptor. PIAS4-catalyzed K187 SUMOylation (non-TRIM ligase) stabilizes ACE2 to suppress K48-linked ubiquitin and autophagic degradation. Stabilization enhances SARS-CoV-2 entry. SENP3-mediated deSUMOylation, on the other hand, facilitates ACE2 degradation through TOLLIP, decreasing infection (Jin et al., 2022). While TRIM28 (Ren et al., 2024) and TRIM32 (Balakrishnan et al., 2025) modulate viral proteins, PIAS4 (Jin et al., 2022) illustrates how SUMOylation of host factors also shapes infection susceptibility. Collectively, these findings underscore the functional versatility of SUMOylation: TRIM28 facilitates viral immune evasion by modifying the N protein, TRIM32 imposes a replication blockade by inhibiting NSP14, and PIAS4 modulates host entry dynamics. Ligase-substrate specificity suggests that targeting the SUMO pathways selectively is feasible, with promising leads toward viral repression and host protection. These pro-viral and antiviral SUMOylation pathways, spanning spike and N-protein modification, NSP14 restriction, and ACE2 regulation, are integrated in Figure 5.

#### 2.3.3 SUMOylation and long-term pathogenesis: inflammation and autoimmunity

While most studies on SUMOylation focus on its functions in acute infection, recent evidence points to its role in chronic dysregulation of the immune response in long COVID. Thurner et al. (2022) demonstrated autoantibodies to a SUMO1-conjugated form of the RNA helicase DHX35 at lysine 53 in about 10% of adult women with long COVID. Interestingly, the autoantibodies recognized only the SUMOylated species, not the non-modified protein, suggesting that SUMOylation itself is a neoepitope-inducing process (Thurner et al., 2022). This response is distinct from conventional autoimmunity mediated through molecular mimicry or epitope spreading and implies that virus-induced post-translational modifications can directly shatter immune tolerance. While the exact function of DHX35 remains to be determined, its designation as a DEAD-box helicase suggests possible roles in RNA metabolism and immune regulation (Thurner et al., 2022). Notably, these autoantibodies were not detectable in healthy adults or in SARS-CoV-2-infected children, suggesting a possible sex- and age-dependent susceptibility. Whether this reflects a sign of differential SUMOylation dynamics, hormonal effects, or immune regulatory thresholds remains to be determined (Thurner et al., 2022).

In summary, autoantibody detection of SUMO1-DHX35 in long COVID proposes a new link between virus-induced SUMOylation and triggering of autoimmunity. It presents new lines of investigation into post-translational modifications as etiological causes of chronic inflammation and therapeutic opportunities in post-viral syndromes. To synthesize the mechanistic insights described above, Table 3 summarizes the major SUMOylation events involving viral proteins, host E3 ligases, and long-term pathogenic processes associated with SARS-CoV-2.

**Table 3:** Summary of SARS-CoV-2–associated SUMOylation events across viral proteins, host factors, and long-term pathogenic processes.

Viral/host factor	SUMOylation site/enzyme	Mechanism	Functional consequence	Unique insight/context	References
Nucleocapsid (N) protein	Human SUMO machinery (SUMO1/2/3 at K65, primarily)	SUMOylation at three lysine residues (K65 key); enhances self-oligomerization and nuclear translocation	Promotes viral RNA packaging, nuclear-host factor interactions; supports virion assembly and potential immune modulation via nuclear reprogramming	Context-dependent nuclear targeting; graded enhancement of N multimerization for stage-specific roles in replication vs. packaging	Madahar et al. (2023)
Spike protein	SUMO1 (trimerization/release); SUMO2 (cleavage/transmission) at K129/K1269 via conserved SIMs	SUMO1 stabilizes trimer for virion release; SUMO2 enhances furin cleavage for cell–cell fusion; cpSIM2 peptide disrupts interactions	Augments spike trafficking, assembly, and transmissibility; cpSIM2 inhibits infection *in vitro* and in hACE2 mice	Differential SUMO1/2 roles create multivalent regulation; peptide-based interference as broad-spectrum therapy	Wang et al. (2025)
Spike RBD (Delta Plus/Omicron)	SUMO-fusion (artificial)	SUMO tagging suppresses amyloid-like fibrils	Reduces aggregation; Omicron more amyloidogenic	Suggests potential anti-aggregation role in neuro-COVID	Zargan et al. (2024)
NSP14 (exonuclease)	TRIM32 (E3 SUMO ligase) at K9/K200	SUMOylation disrupts RNA binding and NSP10 co-factor recruitment; impairs proofreading fidelity	Blocks accurate viral replication; restricts coronaviral proliferation independent of IFN	Intrinsic host restriction pathway; shared across coronaviruses, enabling pan-coronaviral inhibitors	Balakrishnan et al. (2025)
Nucleocapsid (N) protein	TRIM28 (E3 SUMO ligase) at K65	Catalyzes SUMOylation; promotes oligomerization, RNA binding, and LLPS; R203K mutation creates new site	Suppresses innate immune signaling; enhances viral fitness via phase-separated condensates	Proviral cofactor role; peptide disruption restores IFN signaling, highlighting TRIM28 as druggable target for LLPS inhibition	Ren et al. (2024)
ACE2	K187 (SUMO1 by PIAS4; reversed by SENP3)	SUMOylation stabilizes ACE2	Enhances viral entry	DeSUMOylation promotes ACE2 degradation	Jin et al. (2022)
DHX35	K53 (SUMO1)	Virus-induced SUMOylation creates neoepitope; autoantibodies recognize only SUMOylated form in ~10% long COVID cases (adult females)	Triggers autoimmunity via tolerance breach; links to chronic inflammation without molecular mimicry	Sex/age-specific (absent in children/healthy); DEAD-box role in RNA/immune regulation implicates post-viral dyshomeostasis	Thurner et al. (2022)

### 2.4 Glycosylation—a shield for immune evasion and receptor binding

#### 2.4.1 Molecular landscape of spike glycosylation: sites, structures, and functions

Spike glycosylation in SARS-CoV-2 serves complex structural and functional roles. Watanabe et al. identified all 22N-linked glycosylation sites by mass spectrometry and observed a high ratio of oligomannose-type glycans, which differed from host-like maturation. This host mimicry element, to some degree, will ensure immune evasion through the structural stability of the immunogen (Watanabe et al., 2020). In a complementary functional view, Huang C. et al. (2021) demonstrated that removal of N-glycans by enzymatic treatment greatly enhanced S1-ACE2 binding affinity in a wide range of expression systems. Molecular dynamics also observed steric hindrance and electrostatic repulsion between glycans at the RBD-ACE2 interface and showed that glycosylation is a receptor engagement gatekeeper (Huang C. et al., 2021). The results demonstrate an evolutionary trade-off between immune shielding and receptor accessibility. Sanda et al. (2021) added O-glycosylation to the landscape, identifying core-1 and core-2 structures at Thr678 near the furin cleavage site and detecting LacdiNAc and polyLacNAc motifs across several peptides. Their LC–MS/MS-based findings imply that O-glycans may influence proteolytic activation and are spatially poised to regulate S1/S2 cleavage, in contrast to N-glycans that dominate the RBD (Sanda et al., 2021).

Das et al. (2024) identified two distinctive N-glycans at Asn331 and Asn343 as important for ACE2 binding and pseudovirus entry. Mutation of these positions inhibited receptor binding and inhibited IL-6 release from infected cells. Notably, these glycans interacted with host lectins such as Galβ1-4GlcNAc, suggesting a possible role in viral entry and pro-inflammatory signaling, and patient data indicated that spike levels correlated with IL-6 (Das et al., 2024). Wang et al. (2025) employed GaMD simulations to examine the dynamics of N234 and N343 glycans during ACE2 binding. They observed that the glycans dynamically control pocket accessibility in the RBD. While N234 mainly repressed pocket entry in the closed states, N343 exposure elevated pocket accessibility after ACE2 binding, consistent with its multifunctional flexibility across various studies (Cheng et al., 2025). Finally, Neetu et al. (2025) used a 95-lectin array to profile glycosylation of the spike and saw strong binding of virus particles and recombinant spike to lectins that recognize high mannose, fucosylated, and Lewis-type glycans. Their results corroborated the previously identified glycan themes and proposed lectins as possible antiviral leads (Neetu et al., 2025).

**Figure 6:**
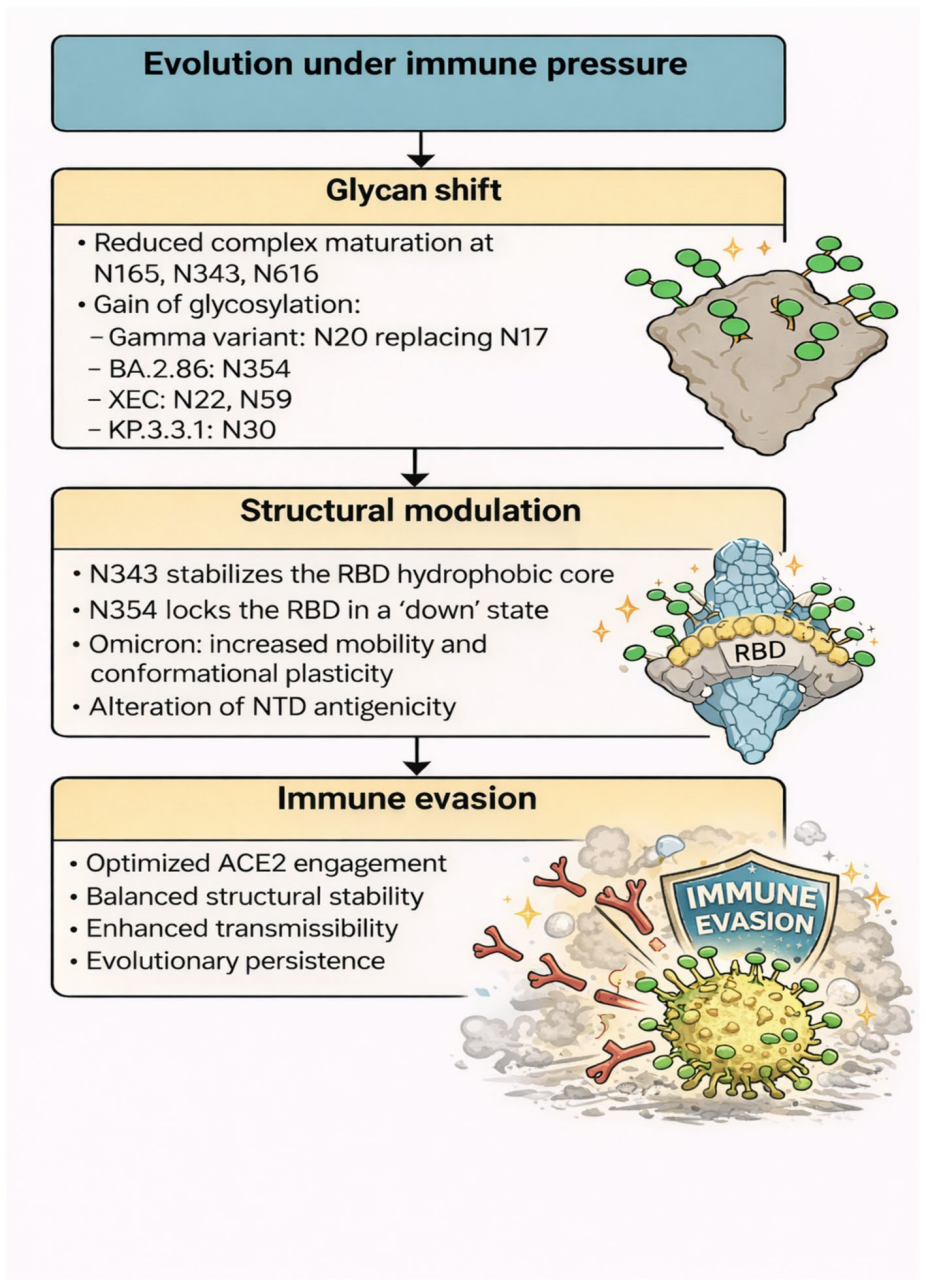
Therapeutic and vaccine strategies targeting conserved spike glycans. Lectin blockade: Host lectins CLEC4G/CD209c bind conserved spike glycans and disrupt RBD–ACE2 engagement, reducing viral entry. Antibody–drug conjugate: A non-neutralizing anti-spike ADC coupled to the STT3A inhibitor NGI-1 induces selective spike deglycosylation in infected cells, exposing neutralizing epitopes. Lectin BOA: The high-mannose–binding lectin BOA cross-links spike proteins into aggregates, neutralizing diverse variants. Vaccine glycoforms: Subunit vaccines enriched for complex-type spike glycans elicit stronger neutralizing and RBD-specific responses than high-mannose variants, underscoring the immunogenic impact of glycoform design.

Collectively, these studies reveal a multi-layered glycosylation landscape on the SARS-CoV-2 spike, with structural roles in folding and shielding (Watanabe et al., 2020; Sanda et al., 2021; Neetu et al., 2025), functional roles in receptor engagement and immune modulation (Huang C. et al., 2021; Das et al., 2024), and therapeutic relevance in modulating pocket accessibility (Cheng et al., 2025). The recurrent emphasis on N343 as a nodal point across structure, receptor binding, cytokine signaling, and drug targeting suggests it is a prime candidate for site-specific therapeutic modulation. These data collectively argue that spike glycosylation is not a passive camouflage but a dynamic, regulatory interface at the heart of viral-host interactions. These emerging immunotherapeutic and vaccine approaches that exploit the spike glycan architecture—including lectin blockade, glycan-editing conjugates, multivalent BOA cross-linking, and engineered vaccine glycoforms—are integrated in Figure 6.

#### 2.4.2 Glycosylation as a driver of immune evasion and variant evolution

The persistent evolution of SARS-CoV-2 variants under immune pressure has made spike glycosylation not just a structural property but a dynamic regulator of viral fitness and immune evasion. DeGlyPHER was used by Baboo et al. (2023) to compare the glycosylation of the historic Wuhan-Hu-1 spike with seven of its top variants and showed partial conservation of N-glycan processing but uniform diminution of complex glycan maturation at sites N165, N343, and N616, key sites for spike function and immune perceptibility. The diminished complexity at these sites may be an evolutionary strategy to reduce immune perception at the expense of receptor binding. Zhang F. et al. (2024) also studied the functional impact of glycosylation, demonstrating that the individual deletion of a particular N-glycan, N343, influenced spike incorporation into virions and pseudovirus infectivity. Although overall glycosylation was necessary for spike function, the N343 glycan alone diminished polyclonal sera’s neutralization susceptibility, acting as an immunomodulatory component in the RBD (Zhang F. et al., 2024). Importantly, post-vaccination convalescent plasma broke this protection, indicating that vaccine-enhanced immunity would neutralize glycan-shielded variants more effectively.

Direct comparison of the Gamma strain to ancestral strains, Pegg et al. (2024) identified that two new N-glycosylation sites in the N-terminal domain of the Gamma spike changed site occupation, where N20 replaced N17 as a result of a T20N mutation. Substitution of the site eliminated NTD-specific antibody binding such as COVA2-17, but not other epitopes (Pegg et al., 2024). This emphasizes how even slight changes in glycosite topology can perturb immunodominant epitopes without overall destabilizing spike antigenicity. Wang D. et al. (2024) also extended this concept to Omicron, demonstrating that modified N-glycosylation patterns at N149 complement spike mutations to increase surface accessibility, particularly in mobile areas such as the RBD and NTD. MD simulations and hydrogen-deuterium exchange indicated increased conformational plasticity for Omicron over D614G, including glycosylation, which plays roles not only in immune evasion but also in epitope presentation and antigenic landscape remodeling (Wang D. et al., 2024).

Glycan site N343 was an evolutionary repeat switch. Ives et al. (2024) reported that, across variants, it preserves the RBD hydrophobic core and allosterically modulates the receptor-binding motif (RBM). Loss caused variant-specific structural changes destabilizing WHu-1, Alpha, and Beta, but not Delta and Omicron, whose RBD stiffness was preserved by compensatory mutations (Ives et al., 2024). This plasticity underscores how glycosylation sites, such as N343, change under modest biophysical stresses, yielding fitness gains without structural instability. In a related study, Watanabe et al. (2020) reported that new glycosylation at N354 within BA.2.86 sublineages reduces infectivity by locking the RBD in a “down” conformation. Importantly, this conformational arrest was competed by heparan sulfate mimetics binding the N354 pocket. N354 glycosylation not only amplified cleavage of the spike and cell-to-cell fusion but also prevented ADCC antibodies, demonstrating a multi-layered immune evasion mechanism, structural regulation, and transmission capability (Liu et al., 2024).

Glycosylation’s impact on the NTD was also shown by Li P. et al. (2025), who studied the T22N and F59S mutant XEC variant. The T22N mutation introduced an additional glycosylation site, reducing neutralization titers in the cohorts. Removing this glycosylation significantly restored neutralization, suggesting it is a causal factor in immune evasion. In addition, glycosylation decreased spike shedding and fusion proficiency, suggesting a structural compromise between antigenic shielding and spike activation (Li P. et al., 2025). Finally, Feng et al. (2025) analyzed the KP.3.3.1 variant and identified an alternative N30 glycosylation site distinct from neighboring glycan patterns. Although structural cryo-EM analysis indicated minimal shape variation in the spike, functional assays demonstrated epistatic interactions between glycosylation and co-mutations, such as F456L and Q493E (Feng et al., 2025). These observations align with the hypothesis that novel variants maximize immune evasion not through extreme structural reorganization, but rather through subtle glycan adjustments combined with stringent receptor-interaction tuning (Feng et al., 2025).

Together, these studies place spike glycosylation at center stage as an active process of evolution that mediates viral fitness, immunological detectability, and structural stability. Although locations such as N343 and N354 are regulatory nodes that connect structural conformation and antibody evasion, recently evolved glycosites, particularly in the NTD, demonstrate how subtle changes can have disproportionate antigenic consequences. Glycosylation, therefore, not only controls spike biology but also maps the evolutionary progression of variants under immunological pressure. These evolutionary, structural, and phenotypic layers of glycan remodeling across variants are conceptually integrated in Figure 6, which summarizes how immune pressure drives glycan shifts, structural modulation, and ultimately immune evasion.

#### 2.4.3 Targeting spike glycans: immunotherapeutic and vaccine implications

The SARS-CoV-2 spike glycoprotein is increasingly recognized as a glycan-rich target for vaccine and immunotherapeutic approaches. Hoffmann et al. (2021) screened in-depth mammalian lectins and determined that CLEC4G and CD209c are strong binders of conserved spike glycans. The lectins directly disrupted RBD-ACE2 binding and elicited strong inhibition of *in vitro* viral entry, consistent with their potential as pan-variant inhibitors owing to the evolutionary conservation of spike glycosylation sites (Hoffmann et al., 2021). Huang H. C. et al. (2021) used an intracellular complementing strategy. By creating an antibody-drug conjugate (ADC) that combines a non-neutralizing anti-spike antibody with the STT3A inhibitor NGI-1, they triggered selective spike deglycosylation in infected cells. This allowed regained access to neutralizing epitopes and increased antibody and vaccine effectiveness against Beta and Alpha variants (Huang H. C. et al., 2021). Their work also linked host STT3A expression to COVID-19 severity and implicated glycosylation as a host susceptibility axis and a viral shield (Huang H. C. et al., 2021). Guseman et al. (2023) and Fan L. et al. (2024) harnessed lectins’ intrinsic antiviral activity to demonstrate *Burkholderia oklahomensis* agglutinin (BOA) to bind spike glycans with nanomolar affinity and cross-link spikes into aggregates, neutralizing all variants tested, including SARS-CoV-1 (Guseman et al., 2023). Compared to CLEC4G/CD209c (Hoffmann et al., 2021), BOA (Guseman et al., 2023) is an extracellular active protein that acts against high-mannose glycans with a multivalent inhibition strategy that is unlike receptor-blocking lectins and intracellular ADCs.

**Figure 7:**
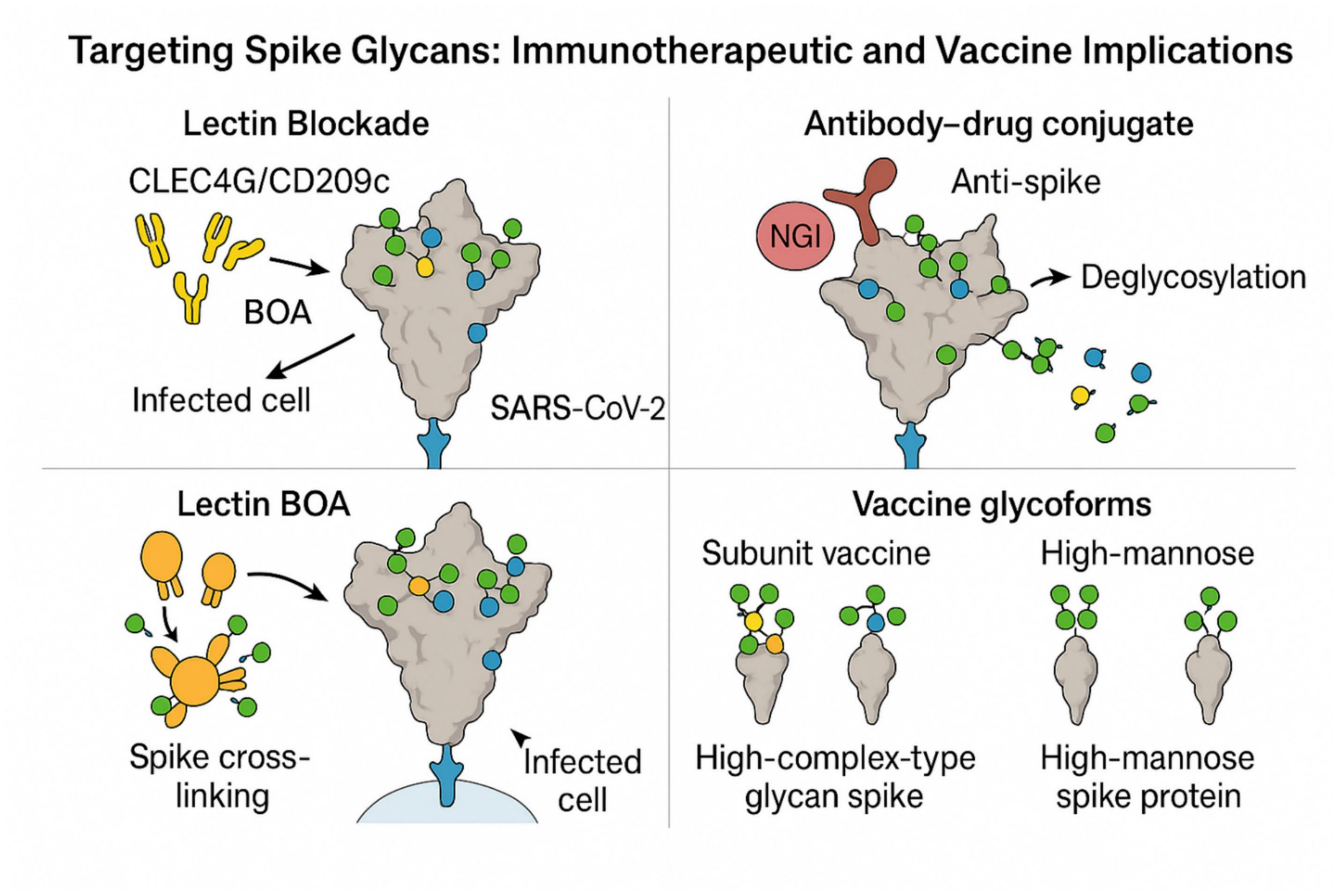
Glycan remodeling under immune pressure drives structural adaptation and immune evasion in SARS-CoV-2. Glycan shift: Variants exhibit reduced complex glycan maturation at key sites (N165, N343, and N616) and recurrent acquisition of new glycosylation motifs across lineages (e.g., N20 in Gamma, N354 in BA.2.86, N22/N59 in XEC, N30 in KP.3.3.1), reflecting convergent evolutionary pressure. Structural modulation: These shifts alter spike architecture, N343 stabilizes the RBD hydrophobic core, N354 restricts the RBD to a “down” state, and Omicron-associated changes increase conformational mobility while reshaping NTD antigenicity. Immune evasion: Collectively, glycan remodeling optimizes ACE2 engagement, balances structural stability with transmissibility, and enhances long-term viral persistence.

Extending beyond viral glycans, Sołkiewicz et al. (2024) also showed that the host IgG glycosylation changes in COVID-19. Increased Lewis^x and core 3 O-glycans during severe illness and decreased T-antigen in convalescents were observed, implicating IgG glycan signatures in disease progression, inflammation, and possibly vaccine response (Sołkiewicz et al., 2024). These results indicate that glycosylation determines not only viral immune evasion but also host immune competence. Finally, Renner et al. (2025) directly manipulated spike-based subunit vaccine glycoforms. They demonstrated, using CHO and insect cell expression platforms, that high-complex-type-glycan spike proteins elicited greater neutralizing Ab and RBD-specific IgG responses in mice than high-mannose or paucimannose equivalents, irrespective of the method of trimerization (Renner et al., 2025). These results emphasize the immunologically significant role of glycoform regulation in vaccine development. Together, these findings highlight spike glycosylation as a tractable and multifaceted therapeutic axis. A consolidated overview of glycan-targeting modalities—including lectin blockade, NGI-1–based ADC deglycosylation, BOA-mediated multivalent neutralization, and vaccine glycoform engineering, is presented in Figure 7.

Collectively, these investigations put spike glycosylation in the proper perspective, as a therapeutic and immunological determinant rather than a biochemical asterism. Lectin blockade, host-glycan interference, and optimization of vaccine glycoforms each demonstrate how glycan-targeted approaches can modulate immune responses and clinical endpoints. The evolutionary conservation of significant sites of spike glycosylation also indicates that they have the potential to serve as broad-spectrum invariant targets. Incorporation of glycoscience into vaccine and therapeutic design thereby has the potential to improve durability and breadth of SARS-CoV-2 interventions. To consolidate the structural, functional, and evolutionary insights discussed above, Table 4 summarizes key glycosylation sites across SARS-CoV-2 spike variants, their mechanistic roles, and their implications for viral entry and immune evasion.

**Table 4:** Mechanistic overview of spike glycosylation sites across SARS-CoV-2 variants and their structural, functional, and immunological consequences.

Viral/host factor	Glycosylation site/type	Mechanism	Functional consequence	Unique insight/context	References
Spike protein	22N-glycans (high oligomannose)	Full glycan map via MS; partial maturation	Structural shielding; folding stability	Oligomannose enrichment	Watanabe et al. (2020)
Spike (S1)	Global N-glycans	N-glycan removal ↑ ACE2 binding; steric gating	Glycans regulate receptor access	Binding–shielding trade-off	Huang C. et al. (2021)
Spike (S1/S2)	O-glycans at Thr678	O-glycans near furin cleavage site	Modulates S1/S2 cleavage	Regulates proteolytic activation	Sanda et al. (2021)
Spike RBD	N331, N343	Required for ACE2 binding; IL-6 induction	Immune modulation + receptor binding	Lectin interaction	Das et al. (2024)
Spike RBD	N234, N343	Dynamic control of RBD pocket accessibility	Allosteric regulation of ACE2 binding	N343 multifunctional node	Cheng et al. (2025)
Spike (global)	High-mannose, Lewis glycans	Strong lectin binding signature	Lectin–glycan antiviral vulnerability	Glycan-based therapeutic entry	Neetu et al. (2025)
Spike variants	N165, N343, N616 ↓ complexity	Reduced complex glycans in variants	Immune evasion via glycan simplification	Adaptive remodeling across variants	Baboo et al. (2023)
Spike variants	N343 deletion	↓ Virion incorporation; ↓ neutralization	Immune-modulatory hotspot	Vaccinated sera overcome escape	Zhang F. et al. (2024)
Gamma variant	N20 (replacing N17)	New glycosite blocks NTD antibodies	Neutralization escape	Minor glycan shift → epitope loss	Pegg et al. (2024)
Omicron	N149 remodeling	Increased RBD/NTD flexibility	Enhanced immune escape	Glycan–mutation synergy	Wang D. et al. (2024)
Spike variants	N343 conserved	Maintains RBD hydrophobic core	Structural stability + immune escape	Compensatory mutation tolerance	Ives et al. (2024)
BA.2.86 sublineages	N354	Locks RBD down; competed by HS mimetics	↓ Infectivity; ↑ immune escape	Multi-layered immune modulation	Liu et al. (2024)
XEC variant	T22N	Additional glycan reduces neutralization	Immune shielding vs. fusion trade-off	Causal role in antigenic escape	Li P. et al. (2024)
KP.3.3.1	N30	Epistatic glycan–mutation interaction	Enhanced immune escape	Fine-tuned antigenic remodeling	Feng et al. (2025)
Spike glycans	CLEC4G/CD209c lectins	Lectins bind conserved glycans; block ACE2	Strong inhibition of viral entry	Pan-variant antiviral lectin	Hoffmann et al. (2021)
Spike intracellular	ADC + NGI-1	Induces selective deglycosylation	Restores neutralizing epitope exposure	Boosts antibody/vaccine efficacy	Huang H. C. et al. (2021)
Spike glycans	BOA lectin	Cross-links spike molecules	Neutralizes all variants	Multivalent extracellular inhibition	Guseman et al. (2023)
Host IgG	Lewis^x↑, core-3↑	Altered host IgG glycosylation	Modulates inflammation & severity	Biomarker potential	Sołkiewicz et al. (2024)
Vaccine spike	Complex-type vs. high-mannose	Complex-type glycans ↑ immunogenicity	Stronger neutralizing Ab response	Guides glycoengineered vaccines	Renner et al. (2025)

### 2.5 Acetylation and epigenetic reprogramming in SARS-CoV-2 infection

#### 2.5.1 Acetylation of viral proteins: structural modulation and host interactions

Acetylation is an important regulatory mechanism that SARS-CoV-2 employs to modulate viral protein function, host interactions, and entry processes. A comparison of the available literature collates the way this post-translational modification controls viral structure and infectivity at different levels. In the N protein, Hatakeyama et al. (2021) demonstrated strong acetylation of host histone acetyltransferases PCAF and GCN5 but not CBP at 12 lysine sites in SARS-CoV-2 but just two in SARS-CoV. Notably, these acetyl sites were localized near RNA-binding and M-protein interaction domains (Hatakeyama et al., 2021). This suggests that SARS-CoV-2 has evolved to leverage host acetylation machinery more intensively, possibly to enhance its RNA packaging, shield replication intermediates from immune sensors, or regulate phase-separated nucleocapsid condensates, a hypothesis worth further structural exploration. Conversely, the study by Vann et al. (2022) targeted the E protein and demonstrated an outward-facing acetylation mechanism. p300-induced E acetylation facilitates high-affinity interaction of bromodomains (BDs) of BRD4, whereas the non-acetylated fragment engages the ET domain (Vann et al., 2022). Bivalent engagement increases viral transcriptional fitness, whereas antagonism through BRD4 inhibitors (e.g., JQ1, OTX015) inhibits infectivity in bronchial epithelial cells (Vann et al., 2022). In contrast to the N protein’s inward acetylation, which is involved in viral assembly regulation (Wang D. et al., 2024), acetylation of E protein promotes bivalent engagement with BRD4, enhancing viral transcriptional fitness, potentially through BRD4’s known role in host transcriptional co-activation (Vann et al., 2022). This suggests an indirect mechanism of host transcriptional subversion, distinct from the more direct structural roles observed for N protein acetylation (Table 5).

**Table 5:** Integrated landscape of viral and host acetylation mechanisms shaping SARS-CoV-2 replication, inflammation, neurobiology, and immune memory.

Viral/host factor	Acetylation site/enzyme	Mechanism	Functional consequence	Unique insight/context	References
Nucleocapsid (N) protein	12 lysines (PCAF, GCN5)	Strong N-acetylation at RNA-binding/M-interaction domains	Modulates RNA binding & N–M interactions; affects phase separation	Higher acetylation vs. SARS-CoV	Hatakeyama et al. (2021)
Envelope (E) protein	p300-mediated acetylation	Acetyl-E binds BRD4 BD; non-acetyl binds ET domain	Boosts viral transcriptional fitness	BRD4 inhibitors suppress replication	Vann et al. (2022)
Spike NTD	9-O-acetylated sialic acids	Spike binds 9-O-Ac-SA as auxiliary receptor	Enhances attachment	Lost in later variants	Petitjean et al. (2022)
Beta variant Spike NTD	9-O-Ac-SA binding	Strong variant-specific acetyl-sialic interaction	Improved attachment	Absent in Omicron	Tomris et al. (2022)
Fusion peptide (FP)	N-terminal acetylation	Supports bilayer insertion	Stronger membrane deformation	Direct link to fusion strength	Miłogrodzka et al. (2025)
TGFBIp (host protein)	K676 acetylation	Correlates with ICU severity	Promotes cytokine storm	Severity biomarker	Park et al. (2020)
Neuronal proteins and histones	>3,800 acetylation sites	Large-scale acetylome remodeling	Neuroinflammation/neuro-COVID	Epigenetic mechanism	Wang Q. et al. (2023)
Macrophages (post-vaccine)	H3K27ac	Persistent promoter acetylation	Long-term trained immunity	Restored by boosters	Simonis et al. (2025)
HDAC6	Deacetylates N protein	Promotes N–G3BP1 interaction	Suppresses antiviral stress granules	HDAC6 inhibitors reduce replication	Mukherjee et al. (2024)
SIRT2	Deacetylates cGAS	Prevents cGAS hyperactivation	Protects against cytokine shock	NAD + rescues lethality	Barthez et al. (2025)

In addition to protein–protein interactions, acetylation also regulates virus–glycan interactions. Petitjean et al. (2022) demonstrated that SARS-CoV-2 was moderately bound to 9-O-acetylated sialic acids (9-O-Ac-SA) and could potentially serve as auxiliary attachment factors in addition to ACE2. Glycoclusters of similar structure effectively inhibited viral binding and entry (Petitjean et al., 2022). Independently, Tomris et al. (2023) also noted that the beta (501Y. V2-1) variant spike N-terminal domain (NTD) exhibited well-defined binding to 9-O-Ac-SA by glycan arrays, ESI-MS, and NMR, but this trait was lost in later variants (Tomris et al., 2023). This temporary acquisition and loss of glycan-binding activity imply that although acetylated sialic acid binding might facilitate entry under certain evolutionary constraints, it is not necessarily preserved through variants that exhibit trade-offs between receptor flexibility and immune evasion. Finally, Miłogrodzka et al. (2025) investigated the biophysical role of N-terminal acetylation of the fusion peptide–membrane interaction. While the unacetylated fusion peptide was unable to bind lipid bilayers, the acetylated one exhibited extremely high affinity toward liquid-ordered cholesterol-rich domains and resulted in excessive membrane thickness and structural alterations (Miłogrodzka et al., 2025). This observation introduces a new twist: acetylation not only controls protein–protein and protein-glycan interactions but also directly enables membrane interactions, a process central to viral entry.

Briefly, acetylation of viral proteins facilitates a wide range of structural and functional modifications, from intracellular assembly to extracellular host interactions. Integration of evidence across the N, E, spike, and fusion peptide proteins positions acetylation as a fundamental mechanism in SARS-CoV-2 biology and an attractive target for intervention.

#### 2.5.2 Host acetylation pathways: epigenetic memory and regulation of inflammation

Host acetylation has emerged as a key modulator of SARS-CoV-2 pathogenesis, with its roles in inflammatory severity, tissue-homing responses, and long-term immune reprogramming. The regulatory mechanism links acute disease mechanisms to chronic epigenetic marks. Park et al. (2020) reported that acetylation of lysine 676 on TGFBIp (K676Ac) is an ICU patient- and COVID-19 severity-independent correlate. In addition to its role as a biomarker, this modification was also functionally implicated in hyperinflammation, as neutralization of TGFBIp reduced the cytokine storm (Park et al., 2020). This supports a model in which specific host protein acetylation actively drives inflammatory amplification rather than passively reflecting disease state.

**Figure 8:**
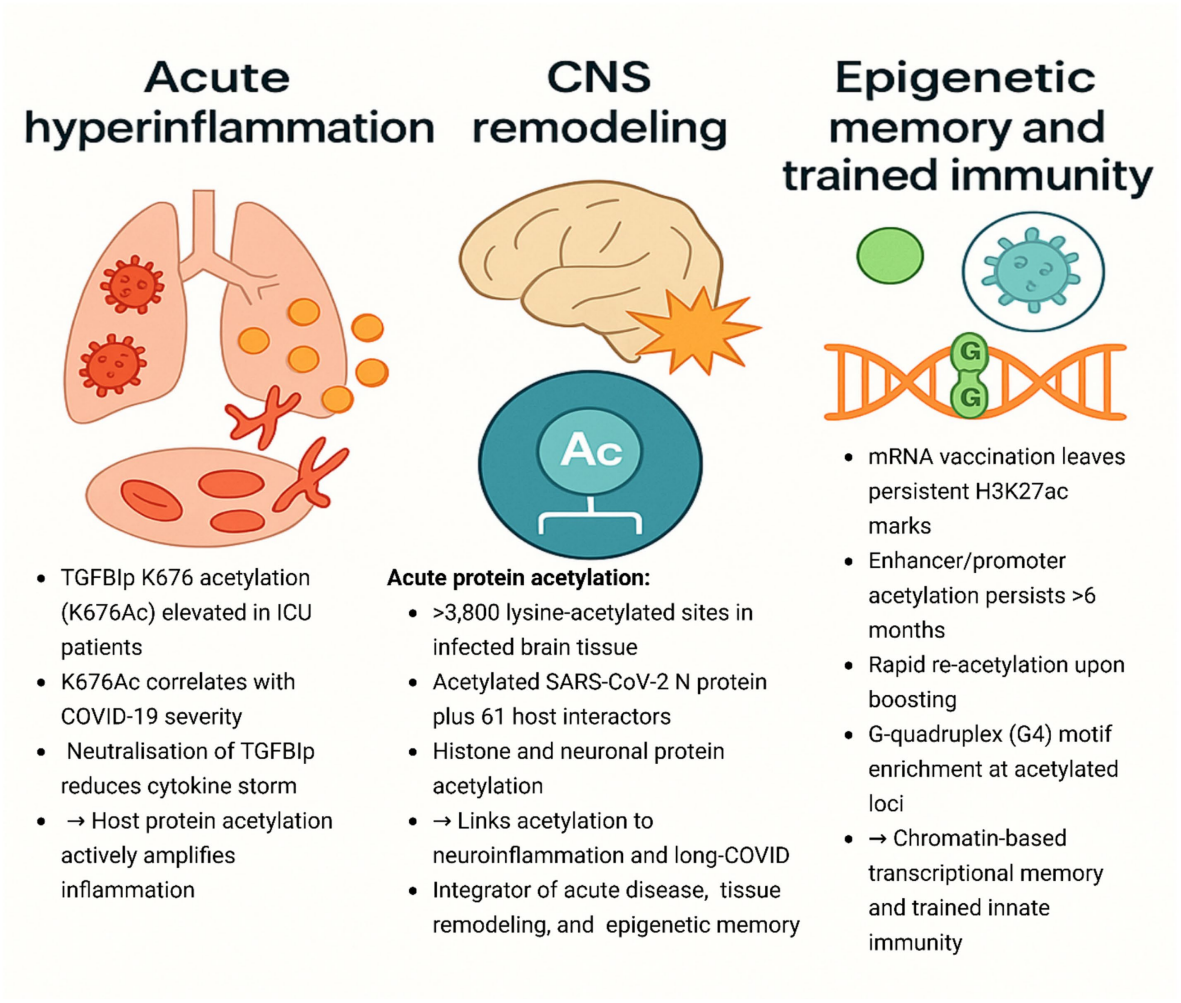
Host acetylation as a regulator of hyperinflammation, CNS remodeling, and long-term immune memory. Acute hyperinflammation: Elevated acetylation of TGFBIp at K676 in ICU patients correlates with COVID-19 severity; neutralizing TGFBIp attenuates cytokine storm, indicating that host protein acetylation actively amplifies inflammation. CNS remodeling: SARS-CoV-2 infection induces widespread lysine acetylation in brain tissue, including histone, neuronal, and viral N-protein acetylation, linking acetylation to neuroinflammation and long-COVID pathology. Epigenetic memory: mRNA vaccination imprints persistent H3K27ac marks at enhancer and promoter regions, stabilized for over 6 months and rapidly restored upon boosting with enrichment of G-quadruplex motifs, supporting chromatin-based transcriptional memory and trained innate immunity.

Expanding the scope to the CNS, Wang Q. et al. (2023) profiled the brain acetylome of SARS-CoV-2-infected K18-hACE2 mice and identified more than 3,800 lysine-acetylated sites, of which a few were in histone and neuronal proteins. An acetylated SARS-CoV-2 nucleocapsid protein was also identified among 61 host acetylated proteins that interacted with viral proteins (Wang Q. et al., 2023). These findings suggest a potential role for acetylation in neuroinflammation and neurological sequelae; however, the K18-hACE2 mouse model exhibits more robust CNS viral replication and neuroinvasion than typically observed in human COVID-19, where direct neuronal infection remains debated and less pronounced. Thus, while the observed acetylomic changes provide valuable mechanistic hypotheses for neuro-COVID and long-COVID pathogenesis, their direct relevance to human disease requires cautious interpretation and further validation in human tissues or more physiologically relevant models. The connection between acetylation and neurologic changes suggests a probable role in neuroinflammation and long-COVID syndrome, complementing the systemic diagnosis of Park et al. (2020) with a tissue-specific aspect. Simonis et al. (2025) explored reprogramming innate immunity via mRNA vaccines by acetylation. They demonstrated that two doses of a vaccine left persistent H3K27ac marks on inflammatory gene promoters in monocyte-derived macrophages, which persisted for more than 6 months and were replenished immediately upon boosting (Simonis et al., 2025). G-quadruplex sequence enrichment in these acetylated sites links chromatin accessibility to long-lived transcriptional memory, providing a mechanism for vaccine-induced trained immunity (Simonis et al., 2025). In sum, these studies tint acetylation as an energetic molecular coat governing the acute and chronic aspects of host–pathogen interaction. While Park et al. (2020) associate acetylation with acute inflammation, Wang Q. et al. (2023) demonstrate organ-specific epigenomic remodeling, and Simonis et al. (2025) show chronic reprogramming of innate immunity. Such a triad charts an array of acetylation impacts from disease to defense to novel leads in diagnostics and immunotherapies in COVID-19 and beyond. A conceptual synthesis of these acetylation-driven pathways, from acute hyperinflammation to CNS remodeling and persistent epigenetic memory, is presented in Figure 8.

#### 2.5.3 Deacetylases as viral co-factors: roles of HDAC6 and SIRT2

Deacetylases have emerged as key players in host–virus interactions during SARS-CoV-2 infection, and HDAC6 and SIRT2 demonstrate how viruses use or hijack host epigenetic regulators to modulate disease processes. Mukherjee et al. (2024) identified that SARS-CoV-2 hijacks HDAC6 through N protein-induced overexpression in infected cells. HDAC6 interacts directly with G3BP1 and N protein, deacetylates the N protein, and promotes its binding to G3BP1 (Mukherjee et al., 2024). This interaction sequesters G3BP1, thereby inhibiting antiviral stress granule formation and promoting viral replication. Functional inhibition of HDAC6, whether genetic or pharmacological, significantly reduced viral replication, suggesting that HDAC6 is a proviral factor hijacked by the virus to evade host resistance (Mukherjee et al., 2024).

Conversely, Barthez et al. (2025) showed that SIRT2 rescues senescent mice from lethal COVID-19 by restricting senescence-associated inflammatory priming by deacetylation of the cytosolic DNA sensor cGAS. In SIRT2-deficient senescent animals, cGAS is hyperacetylated and hyperactivated during infection, leading to hyperinflammation and lethality. Mechanistically, this results from mitochondrial DNA release induced by viral ORF3a protein. Most importantly, NAD + supplementation rescued SIRT2 function and animals, targeting a therapeutically actionable host pathway (Barthez et al., 2025). Although with opposing actions, HDAC6 permissive infection, SIRT2 inhibitory pathology stress both converge on cytoplasmic stress response hubs. HDAC6 targets stress granule dynamics (Mukherjee et al., 2024), whereas SIRT2 modulates innate immune sensing through cGAS. Furthermore, each is induced by distinct viral stimuli: HDAC6 by N protein, SIRT2 by ORF3a-induced mtDNA damage (Mukherjee et al., 2024; Barthez et al., 2025). Collectively, these observations establish a broader context in which SARS-CoV-2 hijacks host deacetylation programs to promote both viral fitness and immune homeostasis. Briefly, HDAC6 and SIRT2 represent two facets of host deacetylation: one commandeered to facilitate viral replication, the other to maintain immune balance against age-related vulnerability. Therapeutically, both enzymes are druggable targets with existing modulators, selective HDAC6 inhibitors (e.g., ricolinostat, citarinostat) have reached clinical trials (primarily in oncology), while SIRT2 inhibitors and NAD + precursors (for sirtuin activation) are available preclinically and clinically, respectively, offering potential for repurposing through selective inhibition of proviral deacetylases (HDAC6) or reactivation of protective ones (SIRT2) in high-risk populations.

### 2.6 Palmitoylation and membrane localization in SARS-CoV-2

#### 2.6.1 Functional importance of palmitoylation in spike and envelope proteins

Palmitoylation, a reversible fatty acylation, is proving to be the key modulator of SARS-CoV-2 structural protein activity, particularly the S and E proteins. Throughout the research, the modification is uniformly associated with increased viral infectivity, protein transport, and virion assembly, but varies in mechanism and effect depending on the protein and context. Mesquita et al. (2021) reported that the spike undergoes rapid, extensive S-acylation at 10 cytosolic cysteines via ZDHHC20 and 9, forming cholesterol-rich nanodomains in the Golgi (Mesquita et al., 2021). This rearrangement of the lipid environment increases fusion competence and virus assembly, indicating that acylation is a structural and functional viral fitness determinant.

Tien et al. (2022) explored the role of four cysteine-rich domains and designated clusters I and II as principal palmitoylation sites. Disruption of these sites suppressed ER-to-Golgi transport, surface expression of spike, and pseudovirus production. Importantly, they highlighted crosstalk between palmitoylation and glycosylation and summarized how multiple PTMs regulate spike maturation (Tien et al., 2022). Li et al. (2022) took a different approach, showing that S cytoplasmic tail palmitoylation is not required for membrane targeting but is essential for syncytia assembly and entry. Using a broader panel of ZDHHC overexpression constructs, they identified several enzymes (ZDHHC2, 3, 4, 8, 20, etc.) associated with spike, suggesting a more distributed enzymatic regulation than previously assumed (Li et al., 2022).

In contrast, Shi et al. (2021) investigated host IFITM3 and showed that its restriction of SARS-CoV-2 is independent of palmitoylation, a notable divergence from its activity against other viruses. In addition, IFITM3 endocytic mutants unexpectedly promoted spike-mediated fusion, suggesting that the host palmitoylation machinery’s antiviral vs. proviral activities are spatially and mechanistically decoupled from viral palmitoylation mechanisms (Shi et al., 2021). Wang Z. et al. (2024) subsequently targeted the envelope protein, describing C40, C43, and C44 palmitoylation (via ZDHHC3, 6, 12, 15, and 20) to be necessary for E protein stability, interaction with other structural proteins (S, M, N), and virus-like particle (VLP) assembly. The palmitoylation was also shown to impact VLP density, associating acylation with virion morphogenesis and infectivity, independent of the spike-mediated fusion process (Wang Z. et al., 2024).

Collectively, these results underscore palmitoylation as a multifaceted regulator of SARS-CoV-2 biology. Spike palmitoylation regulates trafficking, lipid domain organization, and membrane fusion, while envelope palmitoylation is essential for virion assembly and structural integrity. The partial redundancy among ZDHHC enzymes and the varying effects observed with host factors like IFITM3 indicate a complex framework of acylation-mediated regulation, presenting several potential antiviral targets at different phases of the viral life cycle.

#### 2.6.2 Targeting palmitoylation pathways: enzymatic regulators and therapeutic strategies

SARS-CoV-2 spike protein palmitoylation is an essential post-translational modification governing fusion, trafficking, and infectivity. Several enzymatic regulators and therapeutic targets have been identified recently. Zeng et al. (2021) described ZDHHC5/GOLGA7 as acyltransferases that interact with the spike. Notably, knockout was not sufficient to eliminate spike palmitoylation or affect subcellular localization, but it decreased pseudovirus entry, indicating a function beyond direct acylation, potentially as scaffolding complexes. Interestingly, the discovery revealed that APT2 is a spike-interacting depalmitoylase, reflecting a balanced acylation–deacylation regulatory mechanism (Zeng et al., 2021). Ramadan et al. (2022) presented strong pharmacological evidence that two bis-piperazine inhibitors of DHHC9 reduce spike palmitoylation and fusogenic activity. Their observation highlights the irreplaceability of the first five cysteines of the spike tail, positioning DHHC9 as a tractable antiviral target (Ramadan et al., 2022). Contrary to the indirect effects that result from ZDHHC5/GOLGA (Zeng et al., 2021), DHHC9 plays a direct catalytic role (Ramadan et al., 2022).

In a complementary mechanistic insight, Mesquita et al. (2023) demonstrated that SARS-CoV-2 manipulates the zdhhc20 gene’s transcriptional start site, producing an N-terminally extended isoform with 40-fold increased spike acylation efficiency. This virus-driven reprogramming of host S-acylation activity, similarly initiated by unrelated cellular damage, indicates that palmitoylation is dynamically, rather than statically, regulated and is part of broader host stress responses (Mesquita et al., 2023). Collectively, these studies emphasize the pivotal roles of ZDHHC9 and ZDHHC20 as primary palmitoyltransferases, with ZDHHC5/GOLGA7 as backup regulators (Zeng et al., 2021). Therapeutically, enzymatic blockade (Ramadan et al., 2022) and transcriptional control (Mesquita et al., 2023) are viable alternatives. Furthermore, the action of depalmitoylases such as APT2 underscores the promise of dual-target approaches that modulate both palmitoylation and depalmitoylation pathways. Spike palmitoylation is dynamically regulated in the virus-evolved SARS-CoV-2 infectivity process. Its dependence on multiple host enzymes, ZDHHC9, ZDHHC20, and APT2, presents various points of intervention. Strategically targeting these modulators, considering their functional context and timing, promises next-generation antiviral therapy. To summarize the mechanistic and functional insights described in this section, Table 6 provides an integrated overview of palmitoylation events across SARS-CoV-2 proteins and their associated host factors.

**Table 6:** Integrated overview of viral and host palmitoylation pathways regulating SARS-CoV-2 spike function, membrane remodeling, and infectivity.

Viral/host factor	Palmitoylation site/enzyme	Mechanism	Functional consequence	Unique insight/context	References
Spike protein (S)	Ten cytosolic cysteines; ZDHHC20/9	Extensive S-acylation forms cholesterol-rich nanodomains	Increases fusion efficiency and infectivity	Lipid nanodomain restructuring regulates viral fitness	Mesquita et al. (2021)
Spike protein (S)	Cys-rich clusters I	Palmitoylation controls ER–Golgi trafficking and surface expression	Disruption decreases pseudovirus production	Shows palmitoylation–glycosylation crosstalk	Tien et al. (2022)
Spike cytoplasmic tail	Multiple cysteines; ZDHHC2/3/4/8/20	Required for syncytia formation though not membrane targeting	Essential for S-mediated entry	Distributed enzymatic regulation beyond ZDHHC20	Li et al. (2022)
Host IFITM3	–	Antiviral restriction independent of palmitoylation	Mutants unexpectedly promote spike fusion	Divergent from classical IFITM activity	Shi et al. (2021)
Envelope protein (E)	C40, C43, C44; ZDHHC3/6/12/15/20	Acylation stabilizes E & enhances interactions with S/M/N	Essential for VLP formation & virion morphogenesis	Controls virion density	Wang Z. et al. (2024)
Spike–ZDHHC5/GOLGA7	ZDHHC5–GOLGA7 complex	Knockout decreases pseudovirus entry	Reduces viral entry efficiency	APT2 discovered as depalmitoylase of spike	Zeng et al. (2021)
Spike tail (S)	DHHC9 palmitoylation	DHHC9 inhibitors reduce spike palmitoylation & fusion	Produces attenuated/low-infectivity virus	DHHC9 validated as direct catalytic enzyme	Ramadan et al. (2022)
Host ZDHHC20 gene	Virus-induced long isoform	Long isoform increases acylation efficiency ~40×	Enhances spike acylation and infectivity	Dynamic damage-responsive regulation	Mesquita et al. (2023)

### 2.7 ADP-ribosylation—interference with host immunity by the SARS-CoV-2 macrodomain

#### 2.7.1 The multifaceted role of SARS-CoV-2 Mac1 in viral replication and IFN antagonism

SARS-CoV-2 and other SARS-related CoVs encode three consecutive macrodomains in their nsp3 protein. The first of these, known as Mac1 (a MacroD-like macrodomain), is highly conserved across the CoVs family and has the ability to bind and remove (hydrolyze) mono-ADP-ribose (MAR) from target proteins. Mac1 appears to counteract host-induced antiviral ADP-ribosylation, a post-translational modification that forms part of the innate immune response to viral infection. Evidence from various animal models of coronavirus infection demonstrates that Mac1 is critical for viral pathogenesis, establishing it as a key virulence factor and a promising therapeutic target (Alhammad et al., 2021; Brosey et al., 2021). Mono(ADP-ribosyl)ation, or MARylation, is a reversible post-translational modification that regulates protein function through diverse mechanisms (Challa et al., 2021). Multiple lines of converging evidence point to the de-MARylation of STAT1 by the SARS-CoV-2 nsp3 macrodomain as a potential mechanism contributing to the cytokine storm seen in severe COVID-19 cases (Claverie, 2020). Several mechanistic studies reported its bimodal function in MARylation hydrolysis and modulation of the host inflammatory environment, albeit with different interpretations of its role in viral replication and disease pathology. Mukherjee et al. (2024) formulated a hypothesis-driven comparative genomics-based model, assuming that SARS-CoV-2 nsp3 reverses STAT1 MARylation, thereby inhibiting IFN signaling and facilitating cytokine storm in severe COVID-19 patients. Although this initial observation was not based on direct experimental data, it was predictive of a strong mechanistic relationship between Mac1 activity and the hyperinflammatory response, subsequently more unequivocally explored by subsequent research (Claverie, 2020). Alhammad et al. (2021) evidenced by biochemical and structural analysis that SARS-CoV-2 Mac1 is an active adenosine diphosphate-ribosylhydrolase that effectively strips MAR from target proteins with higher catalytic efficiency than SARS-CoV and MERS-CoV homologs. Their study cemented Mac1’s binding to ADP-ribose and revealed conserved binding architectures and broad-spectrum, predictive anti-CoV druggability (Alhammad et al., 2021). Interestingly, despite the three CoVs showing comparable Mac1 functionality, only SARS-CoV-2 showed enhanced substrate turnover, prefiguring evolutionary optimization of this domain to evade immunity.

Further refining the understanding of target specificity, Russo et al. (2021) showed that SARS-CoV-2 Mac1 inhibits ADP-ribosylation elicited downstream of IFN signaling, specifically altering PARP9 and DTX3L but not IFN gene expression itself. This is an important distinction: Mac1 inhibits neither IFN signaling at the transcriptional level but instead reverses its terminal molecular effects (Russo et al., 2021). That understanding reclassifies Mac1 not as an IFN global suppressor but as a selective effector-modulator of post-translational immune responses. That particular activity was measured *in vivo* by Alhammad et al. (2023), who created SARS-CoV-2 mutants deleted for Mac1 (ΔMac1). ΔMac1 SARS-CoV-2 grew normally *in vitro* but was cleared from mice with higher IFN responses and lower pathology than the wild-type virus (Alhammad et al., 2023). Conversely, Mac1 deletion in MERS-CoV and MHV was lethal for the virus. These findings highlight that, unlike other CoVs, Mac1 is not required for SARS-CoV-2 replication but is required for immune evasion and pathogenesis *in vivo* (Alhammad et al., 2023). Mac1, therefore, does not play the role of a replication factor but that of a virulence modulator, a distinction of crucial therapeutic value. To complement these results, Kubon et al. (2024) revealed SQSTM1/p62, the autophagy adaptor, as a new substrate of PARP14-catalyzed MARylation, which was antagonized by SARS-CoV-2 Mac1. The research expanded Mac1’s activity beyond conventional IFN pathways to the regulation of selective autophagy and speculated that viral de-MARylation would inactivate cytosolic protein quality control and immune signal centers (Kubon et al., 2024). In a surprising turn of events, TRIM21 suppressed MARylated p62 degradation, suggesting a complex network of interactions among viral Mac1, host PARPs, and cytosolic adaptors, competing in a molecular arms race for post-translational modification (Kubon et al., 2024).

All in all, these studies reveal that Mac1 is a multifunctional virulence factor: a potent enzymatic tool that has evolved to inactivate host immune defenses, either by disrupting downstream IFN signaling or by directly inhibiting host immune responses. By inhibiting specific MARylated targets such as STAT1, PARP9/DTX3L effectors, and p62, Mac1 is an immunomodulator and mediator of pathogenesis. Notably, differential Mac1 dependence on CoVs implies a single flexibility in SARS-CoV-2 that would be therapeutically desirable. Mac1 inhibitors might not inhibit viral replication per se but would trigger host immunity, providing a new generation of host-targeting antivirals that are resistant to resistance.

#### 2.7.2 Targeting Mac1: structural insights and therapeutic potential

SARS-CoV-2 Mac1, an ADP-ribosylhydrolase domain embedded in nsp3, has become a more prominent structural and functional virulence factor. Progress of the last few years has advanced its conserved fold topology, allosteric plasticity, and immunosuppressive function, promising pharmacological inhibition to restrict virus pathogenesis. But the validity and specificity of Mac1 as a drug target are controversial. Applying this structural finding to evolutionary conservation, Michalska et al. (2020) first obtained high-resolution apo- and ligand-bound Mac1 crystal structures, revealing conformational plasticity and the identification of conserved catalytic water molecules. This study provided the structural foundation for inhibitor development, although without functional correlation (Michalska et al., 2020). In support of this, Hammond et al. (2021) analyzed Mac1 in bat CoV HKU4 and found structural evolutionary conservation and mono-ADP-ribose (MAR) hydrolase activity. Enzyme activity was reduced through mutational destabilization of the binding pocket, confirming functional constraint of the domain across coronaviruses (Hammond et al., 2021).

Building on these insights, Pfannenstiel et al. (2025) described Mac1-targeted pyrrolo-pyrimidine inhibitors. Inhibitors 5c and 6e not only powerfully inhibited Mac1 *in vitro* but also destabilized SARS-CoV-2 replication in IFNγ-rich environments that mimicked the ΔMac1 virus phenotype. Emerging resistance mutations that arose were linked to virus attenuation, further establishing Mac1’s evolutionary bottleneck and therapeutic promise as a high-barrier target (Pfannenstiel et al., 2025). The interferon-dependent context also highlighted an unconventional therapeutic approach: inducing host immunity rather than directly neutralizing the virus. O'Connor et al. (2025) further dissected Mac1’s dual functionality using mutant MHV strains. Catalytic site (D1329A) and binding pocket (N1347A) mutations revealed distinct replication defects: one blocked RNA accumulation, and the other impaired protein production specifically in IFN-competent cells (O'Connor et al., 2025). These results demonstrate that Mac1 regulates both early and late stages of viral replication and that binding and catalytic activities are separately targetable.

In a translational paradigm, Suryawanshi et al. (2025) showed *in vivo* antiviral activity of AVI-4206, a selective inhibitor of Mac1. While poorly active *in vitro*, AVI-4206 inhibited viral replication in human airway organoids and protected mice from lethal SARS-CoV-2 infection, especially under IFN-competent conditions (Suryawanshi et al., 2025). The study set out Mac1 as a candidate immunomodulatory drug target and provided proof-of-concept for pharmacologic inhibition of viral macrodomains. Together, these findings provide an enhanced appreciation of Mac1 as a structurally conserved, functionally multimodal, and therapeutically accessible domain. Structural determinations certify its druggability, whereas functional studies highlight its context-dependent activities in viral replication and immune evasion. Importantly, Mac1 inhibition is most effective when combined with intact IFN responses, making it more of a host-sensitizing antiviral approach than a traditional viral knockout. These mechanistic layers make Mac1 not only an appealing drug target for SARS-CoV-2 but also a template for targeting macrodomains in broader virological contexts. To consolidate the mechanistic insights described above, Table 7 summarizes the major ADP-ribosylation events and Mac1-mediated de-MARylation pathways associated with SARS-CoV-2 infection.

**Table 7:** Key ADP-ribosylation events and Mac1-mediated countermeasures shaping SARS-CoV-2 immune evasion, replication, and antiviral targetability.

Viral/host factor	ADP-ribosylation mechanism/event	Functional outcome	Pathway/host effect	References
Mac1 (nsp3)	Hypothesized reversal of STAT1 MARylation	Inhibits IFN signaling; cytokine storm model	Immune evasion (comparative genomics)	Claverie (2020)
Mac1 (nsp3)	Active MAR hydrolase; enhanced catalytic efficiency	Removes MAR from host proteins	Broad-spectrum druggability; immune evasion	Alhammad et al. (2021)
Mac1 (nsp3)	Inhibits PARP9/DTX3L ADP-ribosylation	Blocks downstream IFN effector function (not transcription)	Selective IFN pathway modulation	Russo et al. (2021)
ΔMac1 SARS-CoV-2	Loss of Mac1 de-MARylation	Cleared rapidly; ↑ IFN; ↓ pathology	Mac1 required for virulence, not replication	Alhammad et al. (2023)
p62 (PARP14 substrate)	PARP14 MARylation reversed by Mac1	Interferes with selective autophagy	PARP14–Mac1–TRIM21 competition	Kubon et al. (2024)
Mac1 structural fold	High-resolution apo + ligand-bound structures	Defines catalytic water and conserved fold	Foundation for inhibitor development	Michalska et al. (2020)
Mac1 evolutionary activity	Bat CoV HKU4 Mac1 retains MAR hydrolase	Mutation reduces activity	Conserved antiviral targetability	Hammond et al. (2021)
Mac1 inhibitors (5c, 6e)	Pyrrolo-pyrimidine inhibitors block Mac1	Impair replication in IFNγ-rich conditions	High-barrier antiviral target	Pfannenstiel et al. (2025)
Mac1 mutants	Catalytic vs. binding-pocket mutations	Impaired RNA accumulation or protein synthesis	Controls early + late replication	O'Connor et al. (2025)
AVI-4206	Selective Mac1 inhibitor active only *in vivo*	Protects mice; active in organoids	Host-sensitizing antiviral (IFN-dependent)	Suryawanshi et al. (2025)

### 2.8 Neddylation—an underexplored modulator of immune homeostasis in COVID-19

Neddylation is a post-translational modification of proteins in which the ubiquitin-like molecule NEDD8 is covalently attached to target substrates, thereby participating in a wide range of critical cellular processes and producing diverse biological outcomes. To date, the most thoroughly studied targets of this modification are members of the Cullin family, which serve as the central scaffolding components of Cullin-RING E3 ubiquitin ligase complexes. By facilitating the ubiquitination and subsequent proteasomal degradation of numerous essential regulatory proteins, these complexes regulate many vital biological function (Wu et al., 2024). Although immune dysregulation in COVID-19 has been thoroughly reviewed, the role of post-translational modifications, such as neddylation, remains unelucidated. Serrano-Maciá et al. (2022), in a landmark study, illustrate that levels of total neddylation are high in COVID-19 patient sera, particularly at the onset of infection. Increased neddylation is associated with increased PBMC activation and cytokine secretion, indicating that neddylation increases hyperinflammatory responses (Serrano-Maciá et al., 2022). Switching significantly, pharmacologic neddylation inhibition suppressed IL-6 and MCP-1 release and triggered global proteomic changes in PBMCs. These results position neddylation upstream of immune tone, poised to affect both the degree and content of the host response (Serrano-Maciá et al., 2022).

In contrast to PTMs like phosphorylation, which are most likely downstream of virus detection, neddylation, in some sense, defines the default activation state of immune cells themselves (Serrano-Maciá et al., 2022). This mechanistic consistency may offer new therapeutic potential. In general, neddylation inhibition can be a novel, selective strategy for immunomodulation in COVID-19, anticipated to suppress pathologic inflammation with minimal compromise of essential antiviral function. More research is needed to define context-dependent effects and time windows for intervention.

### 2.9 Succinylation—an uncharted layer of host metabolic reprogramming

Metabolic rewiring is a characteristic of SARS-CoV-2 infection, but the mechanism of succinylation, a lysine acylation modifying protein charge and conformation, has remained enigmatic. Liu Q. et al. (2022) provide the first global succinyl proteomic analysis in SARS-CoV-2–infected cells, revealing that the virus induces extensive succinylation of TCA cycle enzymes, thereby suppressing mitochondrial metabolism and promoting a glycolytic phenotype. Mechanistically, the viral NSP14 protein directly inhibits SIRT5, the host desuccinylase linking viral replication to metabolic dysregulation (Liu Q. et al., 2022). SIRT5 overexpression, on the other hand, decreased viral load, suggesting that succinylation plays a role as a virus-regulated metabolic switch. Of note, the study also reveals that SARS-CoV-2 structural proteins (N and M) are succinylated at conserved residues, showing functional significance for this PTM during the viral life cycle. Succinylation inhibitors exhibited pharmacologically active antiviral activity, downplaying the therapeutic potential of modulating this process (Liu Q. et al., 2022). Succinylation is a new and amenable interface for immunity and metabolism compared with better-studied PTMs such as phosphorylation (Liu Q. et al., 2022). Overall, succinylation is a dual controller of host and viral proteins, allowing SARS-CoV-2 to rewire cellular metabolism in its favor. This warrants the inclusion of acylation-targeting therapies in host-directed antiviral therapies.

## 3 Non-canonical and emerging PTMs

### 3.1 ISGylation—a battle between host defense and viral evasion

#### 3.1.1 Antiviral roles of ISGylation: inhibiting replication and enhancing immune signaling

ISGylation is a ubiquitin-like post-translational modification in which ISG15, a protein strongly induced by type I interferons and structurally similar to a di-ubiquitin molecule, is covalently conjugated to lysine residues on target proteins (Tecalco-Cruz and Zepeda-Cervantes, 2023). ISGylation occurs primarily as mono-ISGylation (single or multiple on different lysines), although mixed ubiquitin-ISG15 chains have been reported (Xu et al., 2015; Fan et al., 2015). This modification often increases protein stability by competing with ubiquitination and inhibiting proteasomal degradation; for example, proteasome inhibitors can enhance ISGylation levels in IFN-stimulated cells (Liu et al., 2003). ISGylation plays a critical role in innate immune responses induced by type I interferons, contributing to host defense against RNA, DNA, and retroviruses by inhibiting viral particle release, hindering replication, and regulating viral incubation periods through covalent modification of host and viral targets (Zhang et al., 2021). However, viral countermeasures frequently include de-ISGylating enzymes (e.g., papain-like proteases in coronaviruses) that remove ISG15 conjugates to evade these antiviral effects (Liu G. et al., 2021; Gold et al., 2022).

Recent research identifies how ISGylation inhibits SARS-CoV-2 at multiple levels, from the degradation of vital viral components to the regulation of host antiviral responses. Zhu J. et al. (2024) showed that HERC5-catalyzed ISGylation of SARS-CoV-2N protein at four lysine residues (K266, K355, K387, and K388) prevents N oligomerization and RNA replication of the virus. Interestingly, this includes N phosphorylation, which renders it vulnerable to ISGylation (Zhu J. et al., 2024). But the viral PLpro protease behaves in the opposite manner, de-ISGylating ISG15, suggesting an active interaction between host resistance and viral evasion (Zhu J. et al., 2024). A surprising feature of this work was that it placed ISGylation in the regulation of the higher-order assembly of viral proteins, a previously underappreciated role. A companion study by Bang et al. (2024) identified a specific ISGylation site (K261) on the dimer interface of the N protein. They demonstrated that ISGylation at this residue decreases dimerization and renders the virus sensitive to IFN-β. The relevance of this *in vivo* was assessed using a K261R mutant virus, which was resistant to type I IFN (Bang et al., 2024). Both studies converge on the same target nucleocapsid but highlight different facets: while Zhu J. et al. (2024) emphasize oligomer suppression and phospho-dependence, Bang et al. (2024) link ISGylation to antiviral cytokine sensitivity through structural disruption. This reinforces the idea that ISGylation operates through structurally and immunologically distinct but synergistic mechanisms.

Expanding the spectrum of ISGylation targets, Hou et al. (2025) identified nsp8 as a direct target of HERC5. ISGylation targeted nsp8 for proteasomal degradation, disrupting viral replication across different variants, including Omicron sublineages. The study exquisitely illustrates how ISGylation promotes protein clearance compared to N (Hou et al., 2025). Interestingly, PLpro once more acts negatively by reversing ISG15 conjugation, thereby serving as a cross-viral antiviral intervention against numerous ISGylation targets. In addition to viral protein degradation, ISGylation regulates innate immune perception. Liu G. et al. (2021) first demonstrated that ISGylation of the caspase activation and recruitment domains (CARDs) of MDA5 at specific lysine residues promotes its oligomerization and activation, thereby triggering antiviral cytokine production against diverse RNA viruses, including coronaviruses; notably, this activation is directly antagonized by SARS-CoV-2 papain-like protease (PL^pro^) through de-ISGylation. Building on this mechanism, Sarkar et al. (2025) demonstrated that ISGylation of the cytoplasmic RNA sensor MDA5 at K23 and K43 is responsible for oligomerization and the generation of antiviral cytokines. MDA5-KR mutant mice phenocopied the MDA5 knockouts with defective cytokine induction and enhanced viral susceptibility (Sarkar et al., 2025). Together, these studies establish ISGylation as a critical licensing step for MDA5-mediated innate immunity, with Liu G. et al. (2021) providing the initial cell-based mechanistic insight (including SARS-CoV-2-specific evasion) and Sarkar et al. (2025) extending it through *in vivo* validation across RNA viruses.

In another dimension of immune enhancement, García-Arriaza et al. (2025) evaluated ISG15, both wild-type and ISGylation-deficient as an adjuvant in MVA (Modified Vaccinia Ankara)-based vaccines against SARS-CoV-2. ISG15 potentiated isoform-dependent NK and myeloid cell recruitment and augmented antigen-specific T cell responses (García-Arriaza et al., 2025). Similar immunostimulatory effects were observed when ISG15 was used as an adjuvant for Zika virus antigens in the same MVA platform, indicating that these adjuvant properties extend beyond SARS-CoV-2 to other viral pathogens. The observation that ISG15, with diminished conjugation activity, still exhibits immunostimulatory activity indicates that its action is not limited to protein modification, as it also behaves as a paracrine cytokine. Additional information is provided by Hsu et al. (2025), who reported that coronavirus-defective viral genomes (DVGs) selectively modulate ISG15 and IFNβ expression. DVGs stimulated ISG15 expression and inhibited viral replication via type I IFN mechanisms (Hsu et al., 2025). Although indirect, this research suggests that ISGylation is part of a broader feedback loop involving non-coding viral elements and innate immunity.

Together, these works pinpoint ISGylation as a two-pronged antiviral defense. Direct viral replication blocking via protein degradation or functional disruption (e.g., N and nsp8), and immune signaling stimulation via host sensors (e.g., MDA5) are the two prongs. The multi-applicability of PLpro as an ISGylation inhibitor suggests an evolutionary arms race, whereas developments in vaccine and DVG research uncover more significant immunomodulatory features of ISG15. These developments position ISGylation not only as a defense mechanism but also as an adjuvant platform and a drug target worthy of further investigation for emerging viral pathologies.

#### 3.1.2 Viral antagonism via PL pro: subverting ISGylation for immune escape

ISGylation is a crucial post-translational defense mechanism against viral infection. SARS-CoV-2 acts against this strategically through its papain-like protease (PL^pro^), specifically de-boning ISG15 from host and viral proteins. Recent research has elucidated unique yet complementary aspects of this antagonism. In a landmark study, Shin et al. provided the first biochemical, structural, and functional characterization of SARS-CoV-2 PL^pro^, demonstrating its strong preference for cleaving ISG15 (over ubiquitin chains), its contribution to de-ISGylation of IRF3 upon infection, attenuation of type I interferon responses, and the antiviral effects of PL^pro^ inhibition with GRL-0617 in infected cells (Shin et al., 2020). Building on this foundation, Hammond et al. (2021) revealed the SARS-CoV-2 PL^pro’s^ binding and substrate specificities through high-resolution crystallography and solution-state NMR. They showed that PL^pro^ binds human ISG15 and K48-linked di-ubiquitin (K48-Ub) with a two-domain recognition process (Wydorski et al., 2023). Of note here is that PL ^pro^ is seen to bind ISG15 with nanomolar affinity by leveraging the unique interface energetics of its N- and C-terminal UBL domains (Wydorski et al., 2023). Such module-based recognition not only accounts for the specificity of PL^pro^ for ISG15 versus ubiquitin in SARS-CoV-2, but also implies other druggable surfaces that may be targeted to block deISGylation activity specifically. The study redefines PL^pro^ not as a passive deconjugating enzyme but as a sophisticated molecular discriminator that is capable of separating and selectively recognizing host immunity regulators (Wydorski et al., 2023).

Rhamadianti et al. (2024) conducted functional validation by demonstrating that PLpro facilitates deISGylation of the viral N protein. Their findings showed that HERC5-induced ISGylation of N at lysine 374 (K374), which is a conserved position in SARS-CoV and MERS-CoV, inhibits N oligomerization and affects viral genome packaging (Rhamadianti et al., 2024). Importantly, SARS-CoV-2 PL^pro^ actively deISGylates K374 ISG15 and, as a consequence, facilitates N self-association and replication (Rhamadianti et al., 2024). Functional assays also demonstrated that knockdown of ISG15 increases viral replication, whereas overexpression of ISGylation factors suppresses it partially (Rhamadianti et al., 2024). This work brings the results of Wydorski et al. (2023) to life by demonstrating how structural selectivity is conveyed into replication fitness at the level of a key viral protein.

Collectively, these studies beginning with the pioneering work of Shin et al. and extending through structural and functional insights, position PL^pro^ as a well-regulated immune-evasion enzyme, with its structural integrity (Wydorski et al., 2023) alongside its functional effect on viral fitness (Rhamadianti et al., 2024). Their overlap provides the rationale for targeting PL^pro’s^ deISGylation activity as an antiviral strategy.

### 3.2 Disulfide bonds—structural locks in the SARS-CoV-2 spike protein

Disulfide bridges play a pivotal role in ensuring SARS-CoV-2 spike (S) protein stability, not only for proper folding and receptor binding but also for modulating dynamic conformational changes essential for viral entry, immune escape, and assembly. Recent work has offered a multifaceted perspective on covalent bridges as molecular pivot points, structural regulators, and even redox sensors across different phases of the viral life cycle and among spike variants. Grishin et al. (2022) provided the most straightforward mechanistic insight by rigorously challenging the function of disulfide bridges in the RBD of the spike. Molecular dynamics simulations and biophysical assays identified that disruption of four conserved disulfide bridges severely destabilized RBD structure, lowered its thermal stability, and disrupted ACE2 binding by >100-fold (Grishin et al., 2022). Interestingly, RBDs with disulfide disruption showed higher flexibility in surface loops (especially residues 456–490), destabilizing the integrity of the receptor-binding interface. In addition, chemical reduction of the disulfides by DTT or TCEP suppressed viral replication in cell assays, indicating that disulfide bonds are structural, but not solely structural, constraints for receptor binding and infectivity (Grishin et al., 2022).

This structural necessity was reinforced by Prahlad et al. (2021) and Yamaoka et al. (2022), who developed bacterial and wheat germ cell-free expression systems, respectively, for the production of recombinant RBD with intact disulfide bonds. Effective disulfide formation (using CyDisCo, ERO1α, or PDI supplement) was needed in both systems to achieve active RBD that bound ACE2 and patient antibodies. These studies not only confirm the biochemical significance of disulfide bridges but also demonstrate their value for high-throughput antigen production and neutralizing antibody assays. In addition to folding and assembly, disulfide bonds also regulate the conformational states of the full-length spike. Qu et al. (2022) engineered artificial disulfides (e.g., “x3” bond) to trap SARS-CoV-2 spike in rare “locked” pre-fusion conformations. Structural analyses revealed that these locked forms constrain RBD mobility and are favored in acidic compartments during viral assembly and egress (Qu et al., 2022). The locked states disappear upon pH shift to the extracellular environment, triggering transitions to receptor-accessible forms. Interestingly, Zhang et al. (2023) extended this approach to SARS-CoV-1 and showed that engineered disulfide bonds adopt identical locked conformations across lineages, revealing conserved structural dynamics under covalent constraint. These results imply that conformational regulation by disulfides can be one route for controlling spike activation and immune recognition (Zhang et al., 2023). Lastly, Reinke et al. (2024) focused on another viral protein, Mpro, and showed that oxidative stress induces disulfide and NOS/SONOS bond formation in the active site to inhibit protease dimerization and catalytic activity. By extending redox regulation beyond the spike, it also brings disulfides into the limelight as viral sensors of the host oxidative milieu with relevance for viral latency, replication timing, and drug design.

In summary, SARS-CoV-2 disulfide bonds play a function beyond their structural static role. They are redox-regulated determinants of protein conformation, activity, and immune recognition. The combined data from structure, biochemistry, and synthetic engineering studies suggest that disruption of disulfide integrity by reductive stress, small molecules, or engineering could offer new antiviral promise. Additionally, disulfide-mediated locking-domain conservation across coronaviruses provides the prospect of broad-spectrum treatments that stabilize non-coalescent spike conformations or inhibit redox-dependent viral enzymes.

### 3.3 Myristoylation—a hidden driver of SARS-CoV-2 infectivity and egress

While SARS-CoV-2 research has focused on viral entry and replication, mounting evidence indicates that N-myristoylation, the lipid modification catalyzed by host N-myristoyltransferases (NMTs), plays a central role as an autoregulator of virion infectivity and egress. Through computational analysis, Jakhmola et al. (2021) demonstrated that there are recurring mutations in structural proteins (S, M, and E) that establish or destroy motifs for N-myristoylation and PKC phosphorylation. These sequence changes not only influence conformational changes in viral proteins and receptor interactions (e.g., the RGD-integrin interaction of S), but also disrupt host signaling cascades, such as PKC, Src, and NF-κB, that regulate endocytic entry and immune signaling (Jakhmola et al., 2021). Although lacking functional analysis, this work speculated that structural mutations could hijack post-translational signaling pathways, such as myristoylation, to support viral adaptation and pathogenesis (Jakhmola et al., 2021).

Experimentally validating this hypothesis, Saber et al. (2023b) showed that pharmacological inhibition of NMT by selective inhibitors (e.g., IMP-1088) caused a ~ 90% loss of infectivity of SARS-CoV-2 virions released from human lung and primary nasal epithelial cells. Notably, it did not inhibit viral entry, RNA replication, or the release of total virions. Instead, it disrupted the accurate inclusion of envelope proteins in nascent virions, rendering them non-infectious (Saber et al., 2023b). This myristoylation-regulated release–infectivity decoupling illustrates a critical checkpoint in the virus life cycle and indicates that NMT inhibition is not virostatic but rather a physiologic virucidal action. In addition, this research identified a novel reprogramming of viral exit, in which virions bypass the traditional Golgi-dependent secretory pathway and exit through ER–lysosome intermediates, a route otherwise linked to autophagy and stress-induced transport (Saber et al., 2023b). These observations indicate that NMT action may be essential not only for protein processing but also to provide egress fidelity.

A complementary study from the same group (Saber et al., 2023a) confirmed and extended these findings. Using nanomolar concentrations of IMP-1088, the authors reported durable, cell-tolerant inhibition of SARS-CoV-2 spread, with long-lasting suppression of viral infectivity. Intriguingly, the antiviral effect of NMT inhibition was specific to enveloped viruses such as SARS-CoV-2 and RSV, but not to alphaviruses or vesiculoviruses, suggesting a unique dependency on myristoylation within a subset of RNA viruses (Saber et al., 2023a). This virus-selective vulnerability opens the door for host-directed therapeutics that may resist viral mutational escape, an ongoing limitation of spike-targeted vaccines and monoclonal antibodies. Moreover, the slow reversibility of IMP-1088’s antiviral effect suggests that even transient inhibition of NMTs may have prolonged prophylactic potential (Saber et al., 2023a).

Collectively, these studies—primarily from a single group and initially reported as preprints, point to host myristoylation as a key regulator of SARS-CoV-2 pathogenesis, particularly at the sites of virion maturation and non-canonical exit. Computational predictions for acylated myristoylation sites in blood-borne SARS-CoV-2 variants (Jakhmola et al., 2021) are experimentally confirmed by the decrease in infectivity seen following inhibition of NMT (Saber et al., 2023a). Most importantly, the continued decoupling of infectivity from virion release redefines productive infection in our minds and identifies a reexposure vulnerability amenable to therapeutic intervention. Host-dependence and functional selectivity of the process also contribute to the process’s potential as a mutation-insensitive route to antiviral targeting.

### 3.4 S-nitrosylation—a redox switch for therapeutic targeting of ACE2

S-nitrosylation has now shown promise as an anti-viral host-directed mechanism to modulate SARS-CoV-2 pathogenesis at multiple levels, from receptor binding to systemic inflammatory processes. Two mechanistically different studies have revealed the therapeutic significance of this redox switch. In a highly targeted manner, Liu et al. (2003) demonstrated that site-specific S-nitrosylation of ACE2 using aminoadamantane nitrate compounds inhibits spike binding and virus entry without affecting ACE2 enzymatic function. Their dual-action mechanism of action, initial inhibition of viral E protein ion channels followed by release of NO to shield ACE2 from off-target effects, has a localized action (Oh et al., 2023). *In vitro* and Syrian hamster model efficacy render it a precise, mutation-insensitive antiviral approach (Oh et al., 2023). Conversely, Kim et al. (2023) examined a broader host response with exogenous S-nitrosoglutathione (GSNO) and a GSNOR inhibitor (N6022) in mice challenged with a recombinant spike protein. In this, S-nitrosylation provided multi-pathway protection: inhibition of cytokine storms (e.g., TNF-*α*, IL-6), immune cell infiltration, and vascular leakage and thrombosis, all hallmark characteristics of critical COVID-19 (Kim et al., 2023). Of note, a high disease burden and GSNOR expression in male mice, leading to increased treatment response, revealed a redox sex-specific vulnerability (Kim et al., 2023).

Although divergent in their temporal strategies and molecular targets, the two studies converge on the therapeutic potential of S-nitrosylation. Oh et al. (2023) report targeting early viral blockade at the entry site, whereas Zhang et al. (2021) emphasize clearance of secondary inflammation and thrombotic pathology downstream. Such converging findings are consistent with staging or a combination of S-nitrosylation-based therapies to prevent viral and host tissue injury. Cumulatively, S-nitrosylation is a polyvalent therapeutic approach against SARS-CoV-2 that combines receptor-targeted suppression and systemic immune modulation. Cumulatively, these studies offer a redox-based platform for the rational development of second-generation antivirals.

## 4 Crosstalk, systems integration, and multi-PTM dynamics

The SARS-CoV-2 proteome operates not as a sum of independently modified proteins, but as a system of interdependent PTMs that integrate spatial, temporal, and functional information to modulate viral pathogenesis and immune evasion. This PTM crosstalks with the interplay between phosphorylation, ubiquitination, SUMOylation, glycosylation, acetylation, succinylation, and ISGylation, generating complex signaling logic that governs viral replication, host immune suppression, and pathogenic adaptation. A paradigm of this integration is the N protein, where phosphorylation by SRPK1, GSK3, and CK1 remodels its RNA-binding interface and phase separation dynamics (Stuwe et al., 2024; Yaron et al., 2022), while SUMOylation at K65, catalyzed by TRIM28, enhances oligomerization and LLPS, thereby suppressing innate immunity (Ren et al., 2024). Phosphorylation increases structural flexibility in the nucleocapsid protein, as shown by Loonen et al. (2025) through molecular dynamics simulations. Furthermore, ISGylation of N at K266, K355, and K388 is dependent on its phosphorylation state, suggesting phospho-priming for ISGylation (Zhu J. et al., 2024) a classical PTM hierarchy.

The same inter-PTM interaction is observed in Orf6, which degrades not only TRIM25 (ubiquitination axis) but also blocks nuclear translocation of IRF3 and STAT1 (phosphorylation pathway) (Khatun et al., 2023). This dual functionality illuminates how viral effectors execute degradation and signaling inhibition, controlling host antiviral defense through a multi-pronged strategy. The NSP3 protein, together with IRF3 phosphorylation inhibition and binding to dephosphorylated N, acts as a multi-PTM modulator at the systems level as well (Yang et al., 2025). In viral structural proteins, PTM crosstalk is critical for virus assembly and entry. The spike glycoprotein, for example, exhibits site-specific N-glycosylation at N343 and N234, which not only shields epitopes but also dynamically regulates RBD pocket accessibility in concert with conformational shifts (Cheng et al., 2025). Meanwhile, palmitoylation at cytosolic cysteines (via ZDHHC20/9) affects Golgi retention and lipid raft formation, directly influencing spike fusogenicity (Mesquita et al., 2021; Tien et al., 2022). These modifications may work together: glycosylation governs extracellular stability, while palmitoylation coordinates intracellular trafficking and a layered spatial PTM integration.

In addition to protein–protein interactions, acetylation adds a layer of coordination. The E protein, upon being acetylated by p300, interacts with BRD4 bromodomains, hijacking host transcriptional coactivators (Vann et al., 2022). This is regulated by deacetylases such as HDAC6, which is brought into position by the N protein to facilitate increased deacetylation and repression of stress granules (Mukherjee et al., 2024). These findings point toward acetylation–deacetylation balance as a regulatory node downstream of viral structural engagement. Even immune evasion is built upon PTM crosstalk. SUMOylation of MAVS, induced by SARS-CoV-2 N by recruiting UBC9, competes with ubiquitination and interferes with phosphorylation cascades (Huang et al., 2023). This SUMO–Ub–phospho triad is a viral switch that suppresses IFN-*β* production, demonstrating the combinatorial logic viruses use to take over host signaling. From a systems biology perspective, such PTM dependencies create multi-PTM regulatory hubs. The spike protein, nucleocapsid, ORF6, and NSP3 all integrate multiple PTM axes and influence various pathways. This system’s integration enables temporal modulation: early phosphorylation primes later ISGylation; glycosylation establishes early immune evasion; and palmitoylation coordinates late-stage fusion. In sum, SARS-CoV-2 employs a systems-level PTM network rather than isolated marks to coordinate its infection strategy. Recognizing and targeting these multi-PTM dynamics and interaction hierarchies will be essential to designing resilient, host-directed therapeutics in the face of rapidly evolving viral variants.

## 5 Therapeutic implications and drug discovery

Understanding the multidimensional PTM relationships in SARS-CoV-2 infection not only refines our understanding of viral pathogenesis but also provides significant new avenues for therapy. Post-translational modifications are both viral facilitators and therapeutic liabilities, and they place host-modifying enzymes, PTM-interacting domains, and PTM pattern recognition on the drug-accessible vulnerability plate at many points in infection. Several PTM-focused nodes have been defined as high-value therapeutic targets. For example, phosphorylation of N protein by SRPK1/2, CK1, and GSK3 regulates its phase behavior and RNA-binding activity, a process whose viral replication requirement has been demonstrated (Yaron et al., 2022). Blockage of SRPK1/2 not only interfered with N phosphorylation but also strongly repressed viral spread *in vitro* and is consistent with the possibility that kinase inhibition could knock down various arms of the viral replication cycle in concert (Yaron et al., 2022). Moreover, global kinase inhibitors, such as CK2 or p38 MAPK inhibitors, were antiviral by inhibiting cytoskeletal reorganization triggered by the virus and by reprogramming host signaling (Liu X. et al., 2021), demonstrating the druggability of the global phospho-signaling landscape.

The UPS represents another critical vulnerability. Host E3 ligases such as ZBTB25 and Parkin targeted viral proteases Mpro and PLpro, significantly lowering viral burden (Pellman et al., 2024), whereas Ub-variants (UbVs), designed to suppress PLpro activity allosterically, inhibited immune evasion and replication (van Vliet et al., 2022). Conversely, viral proteins ORF6 hijack the UPS to target TRIM25, a key mediator of RIG-I activation (Khatun et al., 2023). Therapeutic approaches could thus not only inhibit viral deubiquitinases but also stabilize or activate host antiviral ligases, such as through PROTAC-directed targeting of viral interface motifs (Zhu K. et al., 2024).

SUMOylation dynamics, though classically associated with nuclear signaling, have emerged as actionable targets. A peptide inhibitor that interfered with TRIM28–N interaction eliminated nucleocapsid liquid–liquid phase separation (LLPS) and re-activated antiviral signaling (6). Likewise, cpSIM2, a cell-penetrating peptide inhibitor interfering with spike–SUMO interactions, exhibited vigorous anti-SARS-CoV-2 activity in transgenic mice, showing that SUMO–SIM interfaces are druggable nodes in structural assembly and host immune modulation too (7). Glycosylation has been leveraged in two ways: (1) lectin inhibitors like CLEC4G and BOA, evolutionarily conserved spike glycan binders used to block viral entry (Hoffmann et al., 2021; Guseman et al., 2023), and (2) antibody-drug conjugates (ADCs) for intracellular delivery of glycosylation inhibitors to deglycosylate spike and expose neutralizing epitopes (Hoffmann et al., 2021). These glycan-targeting approaches are predicated on the evolutionary conservation of glycosylation sites, which endows variants with broad-spectrum potential.

MeL-STPhos and PhosBERT are models that can make highly accurate phosphosite predictions via meta-learning and protein-language models (Pham N. T. et al., 2023; Li Y. et al., 2024). DE-MHAIPs become predictive by the addition of multi-head attention and LSTM-FCN hybrids (Wang M. et al., 2023), but do not remain interpretable. These assist in the rationale-based prioritization of PTM sites, which enables high-throughput screening and repurposing of known kinase or ligase inhibitors with established pharmacokinetics. A remarkable illustration of antiviral engineering against PTM is the Mac1 of SARS-CoV-2 NSP3 that inhibits mono-ADP-ribosylation to repress interferon signaling (Russo et al., 2021; Alhammad et al., 2023). AVI-4206-like inhibitors recapitulated Mac1-deficient virus phenotype and even safeguarded mice in IFN-competent contexts without impairing replication *in vitro* (Suryawanshi et al., 2025). This demonstrates how targeting viral PTM-removing enzymes can selectively enhance host immunity without inducing resistance (Pfannenstiel et al., 2025). More broadly, therapeutic strategies that account for PTM crosstalk, for instance, how phosphorylation licenses ISGylation (Zhu J. et al., 2024) or how SUMOylation shields MAVS from ubiquitination (Huang et al., 2023), may prove more durable and context-sensitive. Drug combinations that modulate upstream enzymes (e.g., SRPK1 or ZDHHCs), while simultaneously restoring downstream responses (e.g., via interferon or autophagy inducers), could circumvent viral compensatory mechanisms.

Overall, the PTM landscape is a multifaceted therapeutic space wherein each axis of modification, phosphorylation, ubiquitination, SUMOylation, glycosylation, and acetylation is a modular switch governing infection processes. Leveraging this modularity through multi-targeted or systems-based design promises to usher in an age of host-directed antivirals, resistant to viral mutation and sensitive to a wide variety of cellular environments.

## 6 Conclusion and future perspectives

SARS-CoV-2 pathogenesis is regulated by a multilayered network of PTMs beyond linear pathways, encoding a dynamic host manipulation logic. This review emphasizes the cooperative and antagonistic roles that phosphorylation, ubiquitination, SUMOylation, glycosylation, and ISGylation play in viral protein phase separation, nuclear transport, and immune evasion, depending on the context. One of the most intriguing findings of this study is that the N protein is controlled by multiple PTMs, with S176 phosphorylation preceding ISGylation and permitting SUMOylation at K65, forming a combinatorial PTM code that controls RNA-binding activity, LLPS dynamics, and suppression of G3BP1-mediated stress granules. In the same way, spike glycoprotein exploits glycosylation at N343 to cover up neutralizing epitopes, and palmitoylation on cytoplasmic cysteines facilitates membrane fusion and Golgi retention. These observations highlight several PTM-based vulnerabilities. SRPK1/2 inhibition eliminates N phosphorylation and abrogates viral replication, and Ub-variants engineered present inhibit PLpro function and reactivate IFN signaling. Peptides against SUMO-interaction motifs (SIMs) block nucleocapsid LLPS and spike nuclear entry, demonstrating that interfaces between PTMs are druggable targets.

Looking forward, the therapeutic strategy is not only to inhibit individual PTMs but also to inhibit multi-site convergence hubs of PTMs that direct viral replication and immune modulation. PhosBERT, DE-MHAIPs, and other AI models, promising as they are, will need to further mature to replicate emergent PTM network properties and model dynamics between infection stages and host cell types. With rapid viral evolution, in which new strains generate novel PTM motifs or reorganize existing ones, dynamic PTM network analysis and targeting will be critical. By moving the antiviral approach from sequence to system—from specific sites to overall PTM logic we can design next-generation therapeutics that are resilient, adaptive, and less susceptible to mutational escape.
